# Construction and characterization of a genome-scale ordered mutant collection of *Bacteroides thetaiotaomicron*

**DOI:** 10.1186/s12915-022-01481-2

**Published:** 2022-12-17

**Authors:** Heidi A. Arjes, Jiawei Sun, Hualan Liu, Taylor H. Nguyen, Rebecca N. Culver, Arianna I. Celis, Sophie Jean Walton, Kimberly S. Vasquez, Feiqiao Brian Yu, Katherine S. Xue, Daniel Newton, Ricardo Zermeno, Meredith Weglarz, Adam Deutschbauer, Kerwyn Casey Huang, Anthony L. Shiver

**Affiliations:** 1 Department of Bioengineering, Stanford University, Stanford, CA USA; 2 Environmental Genomics and Systems Biology Division, Lawrence Berkeley National Laboratory, Berkeley, CA USA; 3 Department of Genetics, Stanford University School of Medicine, Stanford, CA 94305 USA; 4 Department of Medicine, Stanford University School of Medicine, Stanford, CA 94305 USA; 5 Biophysics Training Program, Stanford University School of Medicine, Stanford, CA USA; 6 Department of Microbiology and Immunology, Stanford University School of Medicine, Stanford, CA 94305 USA; 7 Chan Zuckerberg Biohub, San Francisco, CA 94158 USA; 8 Stanford Shared FACS Facility, Center for Molecular and Genetic Medicine, Stanford University, Stanford, CA USA; 9 Department of Plant and Microbial Biology, University of California, Berkeley, CA USA

**Keywords:** *B. theta*, Transposon mutagenesis, Ordered library, BarSeq, RB-TnSeq, Microbiome, Microbiota, Sphingolipid synthesis, Thiamine scavenging, Cell morphology, Cell growth

## Abstract

**Background:**

Ordered transposon-insertion collections, in which specific transposon-insertion mutants are stored as monocultures in a genome-scale collection, represent a promising tool for genetic dissection of human gut microbiota members. However, publicly available collections are scarce and the construction methodology remains in early stages of development.

**Results:**

Here, we describe the assembly of a genome-scale ordered collection of transposon-insertion mutants in the model gut anaerobe *Bacteroides thetaiotaomicron* VPI-5482 that we created as a resource for the research community. We used flow cytometry to sort single cells from a pooled library, located mutants within this initial progenitor collection by applying a pooling strategy with barcode sequencing, and re-arrayed specific mutants to create a condensed collection with single-insertion strains covering >2500 genes. To demonstrate the potential of the condensed collection for phenotypic screening, we analyzed growth dynamics and cell morphology. We identified both growth defects and altered cell shape in mutants disrupting sphingolipid synthesis and thiamine scavenging. Finally, we analyzed the process of assembling the *B. theta* condensed collection to identify inefficiencies that limited coverage. We demonstrate as part of this analysis that the process of assembling an ordered collection can be accurately modeled using barcode sequencing data.

**Conclusion:**

We expect that utilization of this ordered collection will accelerate research into *B. theta* physiology and that lessons learned while assembling the collection will inform future efforts to assemble ordered mutant collections for an increasing number of gut microbiota members.

**Supplementary Information:**

The online version contains supplementary material available at 10.1186/s12915-022-01481-2.

## Background

The human gut microbiota is a complex community that plays a pivotal role in digestion, colonization resistance, immune signaling, and other health outcomes [1, 2]. The mechanisms by which the members of our microbiota exert their effect remain largely unknown. Tools and resources for genetic analysis of microbiota members could be transformative for the mechanistic investigation of microbe-host interactions, but they must be generalizable and scalable to the diverse members of this critical microbial community. The Bacteroidetes phylum includes many species that are prevalent in mammalian gut microbiotas [3] and play important roles in human health [4–7]. *Bacteroides thetaiotaomicron* (commonly referred to as *B. theta*) is a model organism for the *Bacteroides* genus and is of particular interest due to its ability to metabolize complex polysaccharides [8–11] and its capsule production [12, 13], enabling survival within many environmental niches in the gut [14, 15]. *B. theta* antigens have been linked to host T cell responses, which differ depending on diet [16], and *B. theta* exhibits several connections with host metabolism through lipid [17] and outer membrane production [18]. However, genetic mechanisms underpinning the interactions of *B. theta* with its host remain challenging to study because of the effort and time required to generate targeted gene disruptions [19–21].

Genetic disruption via transposon insertion has enabled the creation of pooled mutant libraries that have been used in combination with deep sequencing for genome-wide fitness assays in many bacterial species. In pioneering work, a library of ~35,000 transposon mutants in *B. theta* facilitated a genome-scale interrogation of genes important for in vitro growth and for in vivo colonization of the mouse gut using insertion sequencing (INSeq) [22, 23]. In a recent technological advance, the inclusion of a unique DNA barcode within transposons transforms the relatively laborious and costly transposon-insertion sequencing (TnSeq) protocol into a simple barcode amplification and sequencing protocol (BarSeq) once the mapping between barcode and insertion location has been established [24]. The resulting reduction in cost and effort dramatically enhances the throughput of pooled screens that map genotype to phenotype [25]. We recently constructed a library of >300,000 barcoded transposon mutants in *B. theta* and characterized the fitness of this pooled library across hundreds of in vitro conditions and during mono-colonization of germ-free mice [26]. These pooled screens provided myriad functional and physiological insights, including specific phenotypes for 516 genes, and validation of mutant phenotypes was greatly facilitated by isolation of mutants of interest. Moreover, isolation of single strains is necessary to uncover mutants with phenotypes that are masked in a pooled population, for example involving secretion of a molecule that is shared among the entire population regardless of genotype [27–29]. To facilitate isolation from a pooled library, we recently developed a protocol based on cell sorting that is effective for even strict anaerobes [30].

Here, we report the creation of a genome-scale, ordered collection of *B. theta* transposon mutants in a streamlined, efficient, and generalizable manner. We first arrayed a progenitor collection of nearly 30,000 potential mutants. We then applied a pooled sequencing strategy to identify the mutant in each well, and re-arrayed selected strains to create a condensed collection of >2500 strains with a single transposon insertion per gene. Next, we used the statistics of our progenitor collection along with a quantitative model of the assembly process to identify the factors that reduce coverage during sorting, providing a template for future optimization. Finally, we characterized the growth dynamics and morphology of each mutant in the condensed library. We found that most mutants in the collection exhibited nearly wild-type growth and cell shape. Among the few outliers, we identified mutants in a gene involved in sphingolipid synthesis and a gene involved in thiamine scavenging that were defective in growth and for which a subpopulation of cells was elongated relative to wild type. Given the substantial time and effort required for the construction of targeted mutants in *B. theta*, our collection and strategy for assembly should serve as an impactful resource to gut microbiome research, particularly for investigating mechanisms of commensal survival and function in the mammalian gut.

## Results

### Construction of a genome-scale ordered mutant collection in *B. theta*

**Fig. 1:**
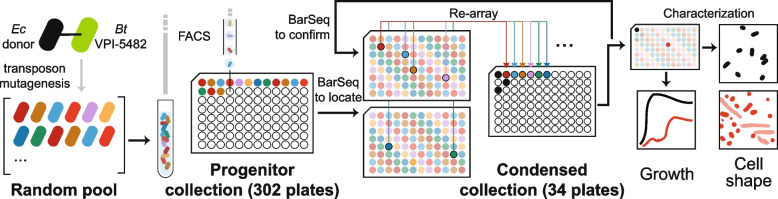
Workflow for the construction of an ordered collection of *B. theta* VPI-5482 transposon mutants. Single cells from a random pool of >300,000 mutants [30] were sorted into 302 96-well plates using FACS to create a progenitor collection. After using barcode sequencing to locate strains within the progenitor collection, representative mutants for individual genes were re-arrayed to create a condensed collection of 34 96-well plates covering >2500 genes. To demonstrate the screening potential of this collection, growth and cell shape phenotypes of each strain were characterized during growth in rich medium (BHIS)

The goal of this work was to assemble a collection of single-transposon insertion strains of *Bacteroides thetaiotaomicron* (*B. theta*) VPI-5482 that covered as many genes as possible and make it available as a resource for the research community. We previously assembled a smaller collection of 40 96-well plates of *B. theta* that included single-insertion strains covering 925 genes [30]. While this resource proved useful for follow-up mechanistic studies [26, 29], the absence of most genes motivated a more extensive collection. Therefore, we sought to expand the coverage of this collection by sorting individual mutants into an additional 262 96-well plates. We used fluorescence-activated cell sorting to isolate single cells and a well-plate pooling strategy combined with barcode sequencing to locate barcodes within the collection (Fig. 1, “Methods”).

**Fig. 2:**
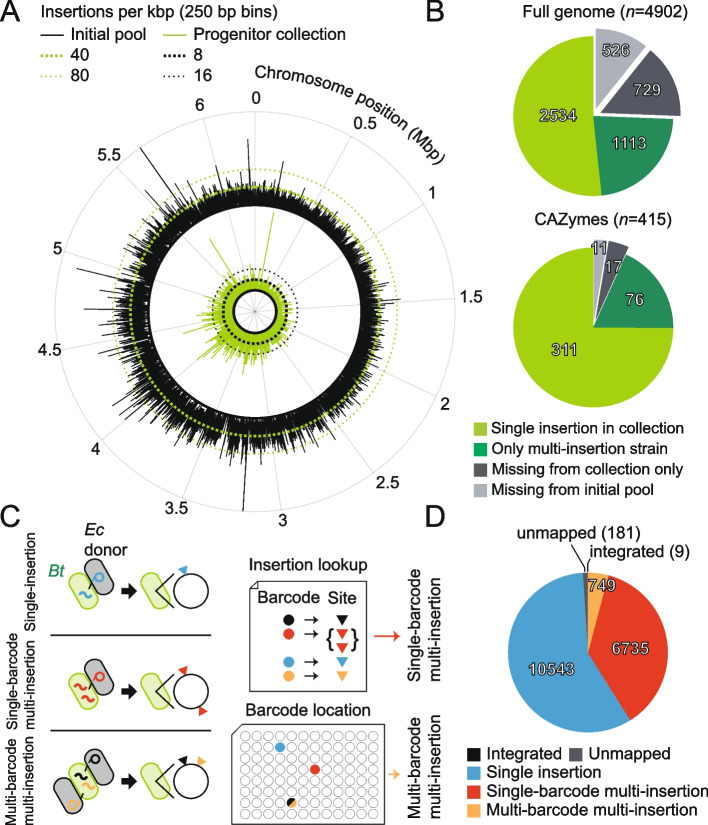
The progenitor collection covers a large fraction of the genome. **A** Patterns of transposon-insertion density across the chromosome were largely similar between the initial pool and the progenitor collection. The insertion density across the chromosome is plotted for the initial pool (~120,000 insertions that map to the genome; outer circle, black) and the progenitor collection (~11,000 insertions; inner circle, green). The number of barcodes detected in the initial pool used for sorting was smaller than the total detected in the original random pool, presumably due to loss of strains during passaging. Insertion density was calculated from the number of uniquely barcoded transposons in 250-bp bins and does not reflect the relative abundance of individual barcodes. Only single-insertion strains were considered when calculating insertion density for the progenitor collection. **B** Coverage in the progenitor collection of the *B. theta* genome (top) and the carbohydrate active enzyme (CAZyme, bottom) functional category. Genes with single-insertion strains were candidates for inclusion in the condensed collection (light green). Some genes were only covered by strains with multiple insertions (dark green), and some had no representative insertions in the initial pool and thus were unlikely to be captured (light gray). Other genes were covered in the initial pool but not captured in the progenitor collection (dark gray); these genes are likely to be accessible with larger collection sizes or with alternative sorting parameters. *n* specifies the number of genes. **C** A schematic connecting the types, causes, and classification of multi-insertion strains. Multiple transposons transferred from the same donor cell during conjugation (red squiggles) are likely to share a barcode, potentially leading to multiple insertions that share a single barcode (red triangles, single-barcode multi-insertion). Transposons transferred to the same cell from multiple donors (orange and black squiggles) are likely to carry unique barcodes, leading to multiple insertions with unique barcodes (orange and black triangles, multi-barcode multi-insertion). Single-barcode multi-insertion strains can be detected within the insertion lookup table as an association between one barcode and multiple insertion sites. Multi-barcode multi-insertion strains can be detected as two barcodes that share the same location in the collection. However, multi-barcode multi-insertion strains cannot be detected in the insertion lookup table, nor can single-barcode multi-insertion strains be detected in the barcode location table. **D** Classification of strains from the progenitor collection according to the number of insertions. Integrated (sequencing past the transposon end mapped to the original plasmid sequence) and unmapped (no sequence information on barcode insertion site) insertion strains were a minority. Most strains were classified as single insertion (light blue). Single-barcode multi-insertion was the second most common classification (light red) and multi-barcode multi-insertion was the third most common (gold)

In the final 302-plate progenitor collection, 2534 genes were covered by at least one single-insertion strain, more than doubling the coverage of the non-essential genome relative to our initial 40-plate collection (Fig. 2A,B). Single-insertion strains in the progenitor collection cover 311 of 415 genes annotated as encoding carbohydrate active enzymes [31] (Fig. 2B), highlighting its potential for investigating carbohydrate utilization. The progenitor collection we report here also expands the scope of gene functions covered relative to existing mutant collections of *B. theta*: our collection contains 1740 unique genes, a previously described non-barcoded mutant collection [23] contains 748 unique genes, and 762 genes overlap between the two collections (Additional file 1: Fig. S1).

The sheer size of the progenitor collection is prohibitive for both screening and distribution, so we re-arrayed isolates from the progenitor collection to assemble a condensed collection. The condensed collection was designed so that each gene of *B. theta* would be represented by one strain carrying a single transposon insertion (Fig. 1, Additional file 2: Dataset S1). We originally re-arrayed into 29 96-well plates, then applied a second round of pooled BarSeq on the condensed collection as a quality check (“Methods”). Analysis of the second pooled sequencing run identified incorrect predictions of strain location and manual errors during the re-arraying process. We re-arrayed into an additional set of 5 96-well plates to correct these issues and complete the condensed collection. In total, the re-arraying step reduced the size of the collection by almost an order of magnitude (from 302 to 34 96-well plates) (Fig. 1), facilitating high-throughput screening and distribution to the research community. The distributions of transposon insertions within coding sequences were essentially uniform in the random pool and progenitor collection, but biased toward the middle of genes in the condensed collection, reflecting our selection criteria at the final step (Additional file 1: Fig. S1, “Methods”). In addition to the genes covered in the condensed collection, the progenitor collection covers 1113 genes that are only covered by strains with multiple insertions (Additional file 3: Dataset S2); these strains are available on request as a resource to the community.

### Identification and exclusion of multi-insertion strains improves collection quality

TnSeq approaches do not explicitly report co-occurrence of insertions in the same genome. Such instances are not a major issue for high-density transposon pools, in which averaging the abundance changes of multiple independent insertions in the same gene minimizes confounding effects of second-site insertions. However, in ordered collections for which one or a few representative strains are chosen to cover each gene, it is important to identify and avoid strains that carry multiple transposon insertions (multi-insertion strains). Traditional approaches for identifying multi-insertion strains, such as Southern blotting, are too low-throughput for genome-scale collections, so we developed reliable predictors of multi-insertion strains using the sequencing datasets derived from the progenitor and condensed collections.

We were able to distinguish between two types of multi-insertion strains based on the barcodes marking each transposon. In one type, recipient cells that received repeated transfers of transposon DNA from a single donor cell would share the same barcode (Fig. 2C, single-barcode multi-insertion). We reasoned that these single-barcode multi-insertion strains could be detected in the RB-TnSeq dataset, as the barcode sequence would be associated with more than one insertion site (Fig. 2C). In the second type of multi-insertion, recipient cells could receive transposon DNA from multiple donor cells and thus have distinct barcodes (Fig. 2C, multi-barcode multi-insertion). We reasoned that multi-barcode multi-insertion strains could be detected as barcodes that co-occur in the same pools of the BarSeq mapping dataset (Fig. 2C).

Applying these two metrics, we found that as few as 58% of strains in the progenitor collection are single-insertion mutants (“Methods”). Furthermore, we found that repeated transfer of DNA from a single conjugative donor cell accounts for the largest population of multi-insertion strains in the random transposon pool used for this study (Fig. 2C,D). We independently verified predicted insertion sites for 9/12 single-barcode two-insertion and 8/10 two-barcode two-insertion strains (“Methods”). While myriad unrelated factors could lower the specificity of the metrics used here to detect multi-insertion strains (e.g., PCR chimeras for single-barcode and cross-contamination of cultures for multi-barcode multi-insertion strains), we conclude that the throughput of this classification scheme make it an important first step toward the identification and exclusion of multi-insertion strains from ordered transposon collections.

### A simple model accurately predicts saturation in the ordered collection

**Fig. 3:**
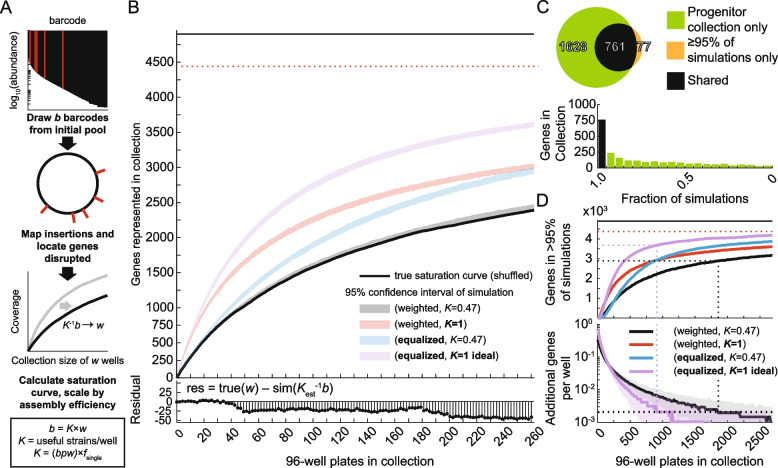
Simulations reveal factors that limit genome coverage in the progenitor collection. **A** Workflow for simulating genome coverage in the progenitor collection. BarSeq was used to estimate the identity and abundance of insertions in the initial pool used for sorting. Relative barcode abundance was used as a probabilistic weight to draw *b* barcodes from the initial pool. The insertions associated with each barcode were located on the genome and compared to the genome annotation to determine the coverage (the number of genes disrupted) of the simulation. To model the coverage of a collection of *w* wells, we estimated the assembly efficiency (*K*, the fraction of wells with a useful barcode): we counted the number of barcodes per well (*bpw*), then factored in the requirement that the barcode had to be associated with a single insertion at a defined genome location. We scaled the collection size in the simulations by *K*^−1^ to account for assembly efficiency. **B** (Top) The experimental saturation curve (black) is plotted along with 95% confidence intervals of simulated saturation curves. The total number of genes (4902) and the total number of genes covered by the initial pool (4374) are shown as black and dashed red lines, respectively. A saturated collection would have coverage that approaches the number of genes in the initial pool. Using the initial pool composition determined by BarSeq and accounting for the assembly efficiency of the collection allowed us to accurately model the true saturation curve (weighted, *K*=0.47). Hypothetical collections with perfect assembly efficiency (*K*=1), unbiased initial pools (equalized), or both resulted in collections with higher coverage. (Bottom) Fit residuals between the true collection (true(*w*)) and the simulated saturation curve sim(*K*_est_^−1^*b*) obtained by scaling the simulation by the value of *K* estimated from the collection statistics. **C** (Top) The overlap between the genes covered in the 262-plate collection and the genes predicted to occur in ≥95% of 250 simulations of the same size is high. While the simulated gene set was largely represented in the 262-plate collection, the larger gene set of the 262-plate collection also contains many genes predicted to be covered at low confidence. (Bottom) Many genes in the collection were present in <95% of simulations (green bars), supporting the conclusion that the 262-plate collection has not reached saturation. **D** Simulations of larger collection sizes predict the requirements for reaching saturation. (Top) The number of genes that occur in ≥95% of 250 simulations is predicted to saturate within 2680 plates only with both ideal assembly efficiency and equalized barcode abundance (equalized, *K*=1; purple line). The horizontal red dashed line represents the total number of genes covered by the initial pool, the saturation limit of any ordered collection. The black and purple dashed lines track collection size statistics for the original (weighted, *K*=0.47) and ideal (equalized, *K*=1) simulations, respectively, with a heuristic practical limit on collection size, as described below. (Bottom) The incremental increase in coverage per well added to the collection can be used to guide the practical size limit of progenitor collections. For example, at a hypothetical incremental efficiency of <2×10^−3^ (horizontal dashed line), an additional 10 96-well plates will only isolate 1–2 additional genes. The collection sizes of both the original (weighted, *K*=0.47; black dashed line) and ideal (equalized, *K*=1; purple dashed line) simulations at this hypothetical limit are represented as vertical dashed lines

We simulated the assembly process to predict the collection sizes needed to achieve saturation in this and future collections. Our simulation uses an estimate of strain abundance from BarSeq data on the initial pool and a parameter for assembly efficiency, *K*, that acts as a conversion factor between the number of wells in the collection and the number of useful strains recovered (Fig. 3A, “Methods”). Using an estimate for *K* of 47% (“Methods”), we found that our model performed remarkably well at describing the true saturation curve (Fig. 3B, top), with a residual of fit of <50 genes (Fig. 3B, bottom).

While we were able to accurately predict the size of our collection, we had mixed results in predicting its composition. Most genes predicted to be covered at high confidence (occurring in ≥95% of simulations) were indeed isolated in the progenitor collection. However, more than half of the genes covered by the progenitor collection had low confidence predictions (occurring in <95% of simulations) (Fig. 3C). These data support the conclusion that the progenitor collection did not reach saturation and highlight the inherent challenge in predicting the composition of non-saturated collections.

We used the simulation to estimate the effect size of two outstanding factors limiting the saturation of the progenitor collection. First, we estimated the effect size of assembly efficiency by setting *K*=1. Second, we estimated the effect size of biases in the abundance of strains by equalizing them in the simulation. Resolving these two factors, either alone or together, significantly improved simulations of the saturation curve (Fig. 3B). Indeed, an “ideal” collection with no strain abundance bias and 100% assembly efficiency would have covered 1177 more genes, an increase of nearly 50% (Fig. 3B).

Finally, we extrapolated to larger collection sizes to determine if a truly saturated collection was achievable for this transposon pool. Given that <50% of the progenitor collection would have been reliably recovered again in a second experiment (Fig. 3C), we used repeated simulations to calculate a saturation curve for genes that would be covered at high confidence (≥95% of simulations). Increasing the collection size to 2667 plates (roughly the contents of a large −80 °C freezer) would only have covered 3166 genes at high confidence (73% saturation). By contrast, an ideal collection would achieve the same coverage at a more practical collection size of 521 plates.

Two conclusions emerge from this analysis. First, simulations can be used to predict statistics of future ordered collections, removing some of the guess work from an inherently random process. Second, the most important challenge for future collections is the identification and elimination of issues that limit assembly efficiency. These points are explored further in the discussion.

### Characterization of growth dynamics reveals a small subset of mutants with reduced growth

**Fig. 4:**
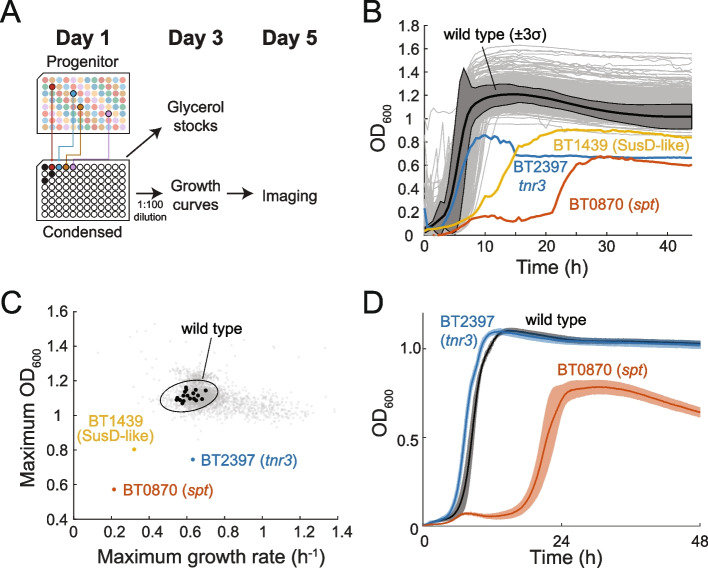
High-throughput screening reveals a small set of mutants with growth defects. **A** Schematic of the pipeline for measuring growth and cell shape phenotypes of the mutants in the condensed collection. **B** Growth in BHIS was similar to wild type for the vast majority of mutants (gray curves). BT0870 (*spt*, sphingolipid synthesis, red), BT2397 (*tnr3*, thiamine scavenging, blue), and BT1439 (SusD-like, yellow) mutants exhibited growth defects. The average of all wild-type growth curves is shown in black with the dark gray shaded region representing 3 standard deviations. **C** Most mutants exhibited similar maximum growth rate and maximum OD_600_ as wild type. Wild-type replicates (black circles) exhibited a similar spread as most of the mutants. All mutants except the outliers described in **B** are shown in gray. The oval indicates 3 times the standard deviations and covariance. **D** After passaging through a colony, the growth phenotype of the BT0870 (*spt*) mutant from the progenitor collection was maintained, while the BT2397 (*tnr3*) mutant reverted to approximately wild-type growth. Solid lines are averages, and shaded regions indicate 1 standard deviation of *n*=6 biological replicates

Previous studies of genome-scale knockout libraries in *E. coli* revealed that only a small subset of gene deletions perturb growth dynamics in rich media [32, 33]. Likewise, *E. coli* cell shape is unaltered by most gene deletions [32, 34, 35]. Nonetheless, unbiased genome-wide screens have identified important components of the pathways controlling growth and cell shape [35, 36]. Given the paucity of similar investigations in most other organisms, our condensed collection provided an exciting resource for discovery regarding *B. theta* growth and cell shape. To this end, we measured the growth dynamics of the condensed collection in a rich medium (BHIS) and used high-throughput imaging [37] to characterize cell morphology of each strain in stationary phase (Fig. 4A).

After re-arraying strains into the condensed collection, we diluted the 48 h-old cultures and regrew each strain in 96-well plates (Fig. 4A). Wild-type cultures were added to empty wells in the condensed collection. Wild-type growth curves were reproducible across plates, with a maximum growth rate of 0.47±0.03 h^−1^ (1 standard deviation) that was reached ~4 h after reinoculation and a maximum OD_600_ of 1.32±0.03 (1 standard deviation) reached after ~13 h (Fig. 4B,C). Most of the growth curves were similar to that of wild type (Fig. 4B), with maximum growth rates and maximum OD_600_ within 3 standard deviations of the mean of the wild-type distribution (Fig. 4C). In addition to the consistency of wild-type growth across plates, sets of technical replicates of each mutant growth curve identified the same outliers with low growth rates and yield, demonstrating that growth behaviors could be effectively compared between 96-well plates (Additional file 4: Fig. S2). Although a small fraction of mutants appeared to reach higher OD_600_ values than wild type, these high-yield outliers were not reproduced in the other replicate and may be due to technical imperfections in a minority of assayed wells (Additional file 4: Fig. S2).

A few mutants had obvious growth phenotypes. The strongest growth defect was associated with an insertion in BT0870, which grew very slowly for ~20 h before transitioning to rapid growth; these growth dynamics were preserved during liquid regrowth from a colony and during further liquid passaging (Additional file 5: Fig. S3, Fig. 4D), suggesting that the later rapid growth is not due to a suppressor mutation. BT0870 (*spt*) encodes a serine palmitoyl transferase essential for the synthesis of sphingolipids in *B. theta* [38]. An insertion in BT2397 had a similar maximum growth rate to wild type but reached a lower maximum OD_600_. BT2397 (*tnr3*) encodes a proposed thiamine pyrophosphokinase involved in scavenging thiamine from the environment [39]. Finally, an insertion in BT1439 had a lower maximum growth rate and did not reach its maximum OD_600_ until 20 h. BT1439 encodes a SusD-like outer membrane protein important during mono-colonization of the murine gut and for the uptake of vancomycin [26].

### High-throughput imaging reveals morphological phenotypes in strains with growth defects

**Fig. 5:**
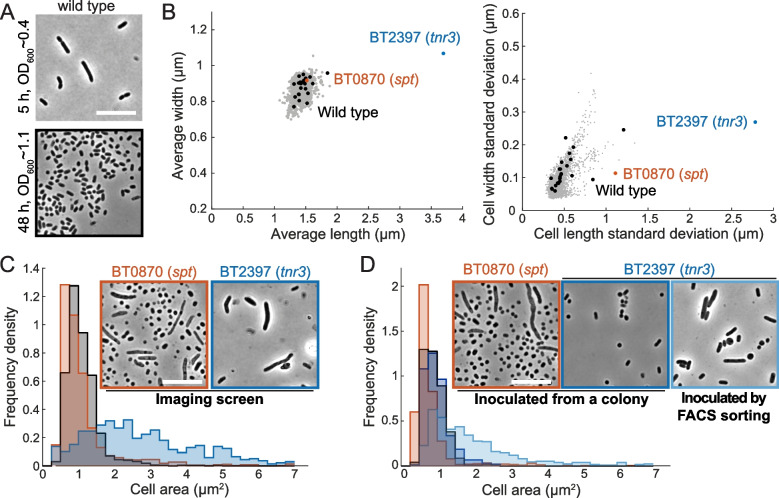
Morphological screening reveals filamentation phenotypes of the BT0870 (*spt*) and BT2397 (*tnr3*) mutants. **A** Wild-type cells become shorter and rounder in stationary phase. Phase-contrast images of log-phase cells (OD_600_~0.4) and stationary-phase cells (OD_600_~1.1). Scale bar: 10 μm. **B** (Left) BT2397 (*tnr3*) mutant cells had higher average length and width than wild-type cells. (Right) BT0870 (*spt*) mutant cells exhibited higher variation in cell length than wild type. All strains except these two outliers are shown in gray, and wild-type replicates are shown in black*. n*>1500 cells per strain. **C** Morphological phenotypes were identified from screening. (Left) A subpopulation of BT2397 (*tnr3*) mutant cells were longer and subpopulations of BT0870 mutant cells were longer or shorter relative to wild-type cells. *n*>1500 cells per strain. (Right) Representative phase-contrast images of each mutant. Scale bar: 10 μm. **D** Morphological phenotypes were retained after single-cell isolation. BT2397 (*tnr3*) mutant cells were no longer elongated after passaging through a colony, but the filamentous phenotype was typically maintained after inoculating individual mutant cells into liquid BHIS using FACS with a gate to select for cells larger than wild type and culturing for 48 h. One representative culture of each strain/condition is shown and the inset shows a representative image of each culture. *n*>400 cells per strain

Using our high-throughput imaging protocol [37], we collected >140,000 images encompassing thousands of single cells for each strain in the condensed collection at the end of the 48-h growth curves in BHIS and used an automated computational pipeline (“Methods”) to segment each cell. During the transition to stationary phase, *B. theta* cells shorten and become rounder (Fig. 5A). As with the growth curves, most strains in the collection had quantitatively similar stationary-phase cell morphology to wild type (Fig. 5B). Nonetheless, the BT2397 (*tnr3*) mutant exhibited larger average cell width and cell length (Fig. 5B), and the BT2397 (*tnr3*) and BT0870 (*spt*) mutants exhibited increased heterogeneity in cell length and/or width. Thus, we focused on these two mutants for further characterization. In the BT0870 (*spt*) insertion, the mode of the distribution of cell areas was shifted to smaller values than that of wild type, and some cells were elongated (Fig. 5C). These cell morphology defects are generally consistent with the known role of *spt* in membrane biosynthesis [38] and of fatty acid availability in bacterial cell-size determination [40].

### BT2397 (tnr3) mutant phenotypes require liquid culturing

The large cell sizes of the BT2397 (*tnr3*) insertion strain were surprising, given that mutants in genes related to the synthesis of thiamine pyrophosphate (BT0652) and the import of thiamine (BT2390, BT2396) were present in the condensed collection but did not display any growth or cell morphology defects. The morphological phenotype of this strain is unlikely to be due to a co-occurring second-site mutation, as an independent strain from the progenitor collection with an insertion in BT2397 (*tnr3*) shared the same elongated cell morphology (Additional file 6: Fig. S4). The BT0870 (*spt*) mutant retained its growth and shape phenotype after passaging through a colony and regrowth in liquid (Figs. 4D and 5D). The BT2397 (*tnr3*) mutant retained its shape phenotypes during liquid passaging from the progenitor stock (Fig. 5C,D, Additional file 6: Fig. S4), but its growth (Fig. 4D) and stationary-phase cell morphology (Fig. 5D) phenotypes were lost upon liquid regrowth after passaging through a single-colony isolation procedure.

To investigate this difference in BT2397 (*tnr3*) outcomes further, we first evaluated the possibility that a mixed population of genetic backgrounds was present in the cryostock and that only the background with wild-type cell shape was stochastically recovered in the small number of colonies isolated. We isolated 64 single colonies after streaking a culture grown from the cryostock on a plate, recovered the colonies in BHIS broth, and measured growth curves and stationary-phase cell morphology. All 64 cultures exhibited wild-type phenotypes (Additional file 6: Fig. S4) and were confirmed via PCR to carry the insertion in BT2397 (*tnr3*). To test whether growth as a colony was responsible for the reversion of the phenotypes, we used FACS to isolate 146 BT2397 (*tnr3*) single cells directly into BHIS broth and grew cultures from them. Eighty-three of these cells were isolated using a gate that selected for mutant cells with approximately wild-type-like size. The other 63 cells were isolated using a gate that selected for mutant cells larger than the average size of the BT2397 (*tnr3*) population. We re-passaged these 146 cultures in BHIS and measured stationary-phase cell morphology. Many of the large-gate and the small-gate cultures exhibited a substantial subpopulation of elongated cells (Additional file 6: Fig. S4), matching our observations from the condensed collection (Fig. 5D). These results indicate that the BT2397 (*tnr3*) mutant indeed has an elongated and heterogeneous shape phenotype in stationary phase and, surprisingly, that growth on an agar surface represses the cell shape phenotype of *tnr3* for at least 2 subsequent passages in broth.

## Discussion

In this study, we assembled a progenitor collection of >300 96-well plates of *B. theta* transposon mutants and condensed it into an ordered collection covering >2500 genes in 34 96-well plates. We demonstrated the suitability of this condensed collection for phenotypic screening by measuring individual growth curves and stationary-phase cell shape in rich media, identifying phenotypes for BT0870 (*spt*) and BT2397 (*tnr3*). We also evaluated the process of assembling an ordered collection and derived a simple and accurate model that identified challenges to overcome to achieve more saturated collections in the future.

Analysis of the *B. theta* collection revealed that achieving saturation of the genome would have been impractical. We identified two main factors that limited the final coverage of the condensed collection. While the high diversity of pooled libraries and deep sequencing minimizes the impact of these issues on pooled fitness assays, our study shows that ordered collections have more stringent requirements that motivate further improvements to transformation protocols. First, biases in strain abundance across the pooled library resulted in the repeated isolation of some barcodes. We expect that an important step toward minimizing biases in strain abundance will be to limit outgrowth of the pooled library before using it as an initial pool during progenitor collection assembly. Second, we ignored strains classified as having more than one transposon insertion, which reduced the number of strains available for re-arraying into the condensed collection. Alternative transformation strategies such as electroporation of in vitro–assembled transposomes [24] could reduce the occurrence of strains with multiple insertions. Further optimization of the pooling protocol and downstream analysis is also likely to improve assembly efficiency. Importantly, the effects of these factors on assembly efficiency could be predicted quantitatively from the statistics of barcodes in the pooled library (Fig. 3), indicating that the necessary collection size, optimal pooling strategy, and other aspects of the assembly of ordered collections can be anticipated for future collections.

Limited returns late in the saturation curve will make 100% saturation of the non-essential genome difficult, even for collections with optimized statistics. Small open reading frames and genes with strong fitness defects will be more difficult to capture from pools by sorting, as they will be represented by fewer strains. Targeted gene disruptions could be used to fill gaps in ordered mutant collections and achieve full saturation. Importantly, the ability to predict the composition of the progenitor collection from the distribution of strains in the pooled library means that genes unlikely to be isolated can be identified ahead of time and targeted disruptions can be prepared in parallel to assembling the random collection. CRISPR editing [41] and CRISPR-guided transposon insertion [42] are promising technologies for targeted mutagenesis in strains without pre-existing tools. CRISPRi is a complementary approach that would also allow for the analysis of essential genes [43, 44]. Nonetheless, we expect that the simplicity of transposon design means that the ability to create ordered transposon collections will outpace adaptation of new targeted mutagenesis strategies in most microbiome strains.

To ensure mostly axenic cultures, we used stringent gating parameters for the cell sorter that were likely to exclude cells with aberrant morphologies, such as the filamented cells expected for cell division and DNA replication mutants [45, 46]. Nonetheless, mutants such as BT2397 (*tnr3*) and cell division mutants with heterogenous cell morphologies [47, 48] can still be isolated using this approach, albeit perhaps at lower frequency. Alternative isolation strategies such as colony picking robots are less likely to bias ordered collections away from abnormal cell shapes, but the effects of colony picking on the parameters controlling assembly efficiency still need to be characterized. Changes to the sorting protocol, for instance relaxing gating parameters or sorting stationary-phase cells that are more homogeneous in size [49], could expand the range of cell morphologies represented in the progenitor collection, and can be included as addendums to the progenitor collection to avoid biasing the primary sort. Since the gut microbiota is composed of species with diverse cell morphologies, the isolation of mutants with aberrant cell shapes will be important for understanding the role of cell shape in gut colonization, biogeography, and niche partitioning.

Nearly all mutants exhibited wild-type–like growth and shape phenotypes in the nutrient-rich medium BHIS, similar to screens of *E. coli* gene deletions in the nutrient-rich medium LB [33, 34]. In addition to reducing strain abundance bias as described above, limiting outgrowth of the pooled library before sorting is likely to reveal more mutants with growth defects in BHIS, as these are the most likely to drop out of the population with repeated passaging. Growth in an environment different from that used to create the pool would also likely reveal environment-dependent growth phenotypes in the collection. Nonetheless, we identified a mutant in BT0870 (*spt*) that exhibited both growth (Fig. 4B–D) and cell shape phenotypes (Fig. 5B,C). A targeted BT0870 (*spt*) deletion was previously reported that exhibited minor growth defects [38]; our growth data, coupled with the observation that this strain was the sole insertion mutant in BT0870 (*spt*) in the original pooled transposon library, suggests that loss of BT0870 (*spt*) function is deleterious and that mutants may be prone to accumulation of suppressor mutations. A more sophisticated genetic system that lowers the risk of suppressor mutations, like regulated expression of *spt* at an ectopic site [44] in combination with a targeted deletion of the endogenous locus [50], may be important for measuring the full set of phenotypes of BT0870 (*spt*), including its essentiality. The filamentous phenotype of some BT2397 (*tnr3*) mutant cells suggests that thiamine pyrophosphokinase plays an important role in cell shape determination in *B. theta*, as it does in fission yeast [51]. Surprisingly, during liquid growth after passaging BT2397 (*tnr3*) through a colony, we observed reversion to wild-type phenotypes. This raises the question of whether creating a targeted deletion of BT2397 (*tnr3*), which typically involves multiple selection steps on agar plates, would capture the phenotype observed here. While the exact nature of this behavior remains to be determined, it illustrates how growth conditions can play a major role in *B. theta* phenotypes. In the future, it will be interesting to screen the condensed collection in diverse media conditions to uncover additional phenotypes, as a previous screen of a CRISPRi library of *Bacillus subtilis* essential-gene depletions uncovered a wider distribution of phenotypes in a minimal medium compared with LB [52].

## Conclusion

The condensed *B. theta* mutant collection we report here will prove useful for addressing numerous phenotypes beyond growth and morphology that are difficult to measure in a pooled format, including the effect of gene disruption on interactions with other species or on metabolite production, as well as mechanistic inquiries about a mutant of interest. Indeed, mutants from our collection have already proven useful for follow-up studies to pooled transposon library screens [26] and to metabolomics measurements [29]. The ordered collections should thus serve as a resource for identifying genotypes important for *B. theta*’s function within microbial communities and in the gut. Moreover, the approach we have developed for the assembly of ordered collections should be generally applicable in any organism for which diverse pooled transposon libraries can be generated, enabling genetics-based interrogation of members of the gut microbiota and the molecular mechanisms through which they impact human health.

## Methods

### Oligos and strains

The transposon-insertion mutants in the condensed collection are listed in Additional file 2: Dataset S1. The genes that are only represented by multi-insertion strains are listed in Additional file 3: Dataset S2. The strains and oligos used in this study are listed in Table S1. Details of the transposon design, transformation, and the original RB-TnSeq mapping experiment were published previously [26].

### Growth conditions

We used BHIS as the base medium for this study, to match growth conditions during the initial construction of the transposon pool [26]. Our BHIS formulation is hydrated Brain Heart Infusion powder (Difco, Cat. #237200) supplemented with 0.2% (w/v) sodium bicarbonate (Fisher bioreagents, Cat. #BP328-500), 0.05% (w/v) porcine hemin (Alfa Aesar, Cat. #A11165), and, in some cases, 0.1% (w/v) L-cysteine (Thermo Scientific, Cat. #A10435.18). In a previous study, we found that exogenous cysteine led to the production of H_2_S by *B. theta* VPI-5482 [30]. With the large volumes required for this approach, the H_2_S levels were enough to saturate our hydrogen sulfide scrubbing column and make working with the collection dangerous. Therefore, for all steps that required growth of the collection in substantial volumes, we did not include cysteine in the BHIS formulation. Cultures were incubated in a custom anaerobic chamber (Coy Laboratories) using an 85% nitrogen–10% carbon dioxide–5% hydrogen anaerobic gas mix (Praxair, Cat. #BI NICDHYC4-K).

### Preparation of the pooled library for sorting of the progenitor collection using flow cytometry

A barcoded transposon mutant library of *B. theta* VPI-5482 mutants was sorted using a fluorescence-activated cell sorting (FACS) machine, as described previously [30] with a few modifications. Cells were sorted into 262 96-well plates in three batches of 60, 100, and 102 plates; the same protocol was used for each batch. These 262 plates were combined with 40 96-well plates that were generated in a pilot experiment [30], leading to the final progenitor collection of 302 96-well plates. All media and plasticware were pre-reduced in an anaerobic chamber (Coy Laboratories) for 3 days before use to eliminate any residual oxygen.

### Addition of medium to the 96-well plates before sorting

Three hundred thirty three microliters of pre-reduced and filter-sterilized BHIS (with no added cysteine) were added to 2-mL 96-deep well plates (Greiner Bio-One, Cat. #780280) using a semi-automated BenchSmart pipettor (Mettler-Toledo) installed in an anaerobic chamber. Each 96-deep well plate with added medium was sealed with a foil seal (Nunc^TM^ Sealing Tapes, Fisher Scientific, Cat. # 232698) and stored anaerobically for 16–24 h at 37 °C before use in the next step of the procedure.

### Outgrowth of the randomly barcoded transposon mutant library

A cryostock of the pooled library (1 mL of OD_600_=1) was thawed in an anaerobic chamber and added to 100 mL of BHIS [26] in a 250-mL Erlenmeyer flask that had been pre-warmed to 37 °C, and the culture was incubated overnight (12–18 h) at 37 °C without agitation. The next day, ~3 h before cultures were transported to the FACS machine, this overnight culture was diluted to an OD_600_~0.1 in 4 mL of BHIS pre-warmed to 37 °C in 4 independent samples. At the same time, BHIS was pre-aliquoted into an additional set of tubes (2 mL per tube) and kept at 37 °C.

### Sorting the pooled library to create the progenitor collection

Immediately before being transported to the FACS machine, log-phase cultures of the transposon library were diluted to an OD_600_ of 0.01–0.05 in pre-warmed BHIS with 10 mM cysteine in a FACS tube (Falcon® high-clarity polypropylene round bottom test tubes, Corning, Cat. #352063) and mixed thoroughly in the anaerobic chamber. An initial culture in a FACS tube was brought out of the chamber and loaded onto the FACS machine to calibrate the instrument and define a gate based on forward and side scatter. After calibrating the machine, a fresh FACS tube of culture and a set of pre-reduced 96-deep well plates were brought to the FACS machine and the fresh culture was used to sort single cells. To preserve the anaerobic environment of the culture, the FACS tube was sealed in an airtight container inside the anaerobic chamber for transportation to the FACS machine, and the FACS tube was disturbed as little as possible after being exposed to oxygen. After single cells were sorted into individual wells of a 96-deep well plate, the deep well plate was lightly resealed with a gas-permeable seal (Excel Scientific Inc., Cat. #BS-25). Batches of 15 96-deep well plates with sorted cells were returned to the anaerobic chamber and new sets of deep well plates with fresh media were transported to the FACS facility as needed to keep the FACS machine in continual operation. Once sorted plates were returned to the anaerobic chamber, the gas-permeable seal was fully sealed using a rubber brayer roller, taking care to seal the edges of the plate to prevent evaporation. The sorted plates were then returned to the 37 °C incubator in the anaerobic chamber. The FACS tube of culture was replaced with a fresh culture every 30 plates. Cultures of the transposon library were kept in log-phase via dilution in the 37 °C incubator throughout the course of the sorting experiment to maintain a supply of log-phase cells for sorting.

### Aliquoting copies of cryostocks of the progenitor collection

The sorted cells were allowed to grow into monocultures over 2 days. Glycerol was added to the cultures, and the glycerol stock was aliquoted into two copies of the library using a semi-automated 96-well BenchSmart pipetting robot inside the anaerobic chamber. Eighty microliters of 50% glycerol was added to each well (final concentration 15% glycerol) and mixed by pipetting up and down. Eighty microliters of the glycerol stock were then aliquoted into two V-bottom 96-well plates (Greiner Bio-One, Cat. #651161). The cryostock plates were sealed with a foil seal and stored at −80 °C. The remainder of the glycerol stock inside the 96-deep well plate was also stored for subsequent pooling steps of the protocol.

### Selection criteria for re-arraying the collection

Only insertions in the middle 85% of genes (positioned after the first 5% and before the last 10%) were considered eligible for re-arraying into the final collection. Multi-insertion strains were not eligible for re-arraying. When more than one single-insertion strain covered the same gene in the progenitor collection, the insertion closest to the middle of the open reading frame was prioritized.

### Re-arraying the progenitor collection

At the beginning of the experiment, erythromycin (Sigma, Cat. #E5389-5G) was added at a final concentration of 10 μg/mL to a bottle of filter-sterilized BHIS (Becton Dickinson) without cysteine that had been left in the anaerobic chamber for 48–72 h to reduce. This selective medium was aliquoted into 8 96-deep well plates (Celltreat, Cat. #229574) using a BenchSmart with a 1000-μL head (Rainin, Cat. #BST-96-1000), sterile filter tips (Rainin, Cat. #30296782), and a 300-mL reservoir (Integra, Cat. #6328). The 96-deep well plates were sealed with a plastic film (Excel Scientific, Cat. #STR-SEAL-PLT), transferred to a second anaerobic chamber using airtight plastic boxes, and stored in a 37 °C incubator inside the anaerobic chamber until inoculation.

Over the course of a day, sections of the progenitor collection were re-arrayed anaerobically using an Eppendorf EpMotion 5073 (Eppendorf, Germany) running EpBlue v. 40.5.3.10 and housed inside an anaerobic chamber. Table S2 contains the settings used for the EpMotion.

Before transfer to the anaerobic chamber, the 96-well V-bottom plates containing glycerol stocks of the progenitor collection were removed from the −80 °C freezer in batches and stored on a bed of dry ice. The lids of each plate were removed, ice was cleaned off, and the lids were sterilized with 70% (v/v) ethanol while the sealed plates were centrifuged in a microplate centrifuge (Fisherbrand, Cat. #14-955-300) for 30 s. After centrifugation, the foil seal was removed from each plate, taking care not to jostle the plate. The clean and sterilized lid was then returned to each plate, and the lidded plates were returned to the bed of dry ice. When the entire batch of plates had been processed in this manner, the plates were transferred into the anaerobic chamber and stored in a safe location on the benchtop.

Next, we inoculated the 96-deep well plates of selective medium with 40 μL of glycerol stock from selected wells of the progenitor collection. Three hundred microliter PCR-clean filter tips (Eppendorf, Cat. #0030 014.472) were used in combination with a single channel 300-μL adaptor (Eppendorf) to transfer glycerol stocks. The pipetting pattern (the set of instructions connecting position in the progenitor collection to position in the condensed collection) was imported into EPBlue as a .csv file after being generated using a Matlab script.

After inoculation, 96-well plates from the progenitor collection were resealed with a foil seal (Thermo Scientific, Cat. #232699) and transferred back to the −80 °C freezer. The 96-deep well plates with freshly inoculated cultures comprising the condensed collection were sealed with a gas-permeable seal (Excel Scientific Inc., Cat. #BS-25) and stored in a 37 °C incubator in the anaerobic chamber to recover for 36-48 h and used to inoculate growth curves and to aliquot copies for cryo-storage.

### Growth curve inoculation

Approximately 48 h post-inoculation, deep well cultures were used to inoculate fresh medium for growth curve measurements and then aliquoted into glycerol stocks as copies of the final condensed collection (glycerol stock storage described below).

First, 198 μL of BHIS without cysteine and without erythromycin was aliquoted across 16 96-well flat-bottom plates (Greiner Bio-One, Cat. #655180) using a BenchSmart with a P1000 head and sterile filter tips. The plates were transferred to the anaerobic chamber along with the previously generated cultures using an airtight sealed container.

The cultures were then used to inoculate fresh medium for growth curves using an EpMotion 5073. Fifty-microliter PCR-clean filter tips (Eppendorf, Cat. #0030 014.430) were used in combination with an 8-channel 50-μL volume adaptor (Eppendorf). Each deep-well culture was used to inoculate 2 96-well flat-bottom plates as replicates for the growth curve measurements. Two microliters of culture were transferred without mixing at the source and with 1 mixing step of 40 μL at the target. The same tips were used, and the source was revisited once, to inoculate a replicate target plate. Table S3 contains the machine settings used for this protocol. To avoid transferring liquid from the intentionally blank wells on each plate, we removed the tips from positions A1, B1, and the other blank well on the plate (Additional file 2: Dataset S1).

For some of the flat-bottom growth curve plates, 2 μL of a culture of wild-type *B. theta* VPI-5482 grown in BHIS without cysteine for 36–48 h were used to inoculate position A1 as a positive control. All flat-bottom 96-well plates were sealed with modified sterile plastic seals, cut to not extend over the edges of the plates, and assembled in a plate stacker (BIOSTACK3WR, Biotek Instruments Inc.) associated with a Synergy H1 microplate reader (Biotek Instruments Inc.) running Gen 5 v. 3.08.01. The plate stacker and the front of the microplate reader were enclosed in a custom-fabricated box along with a thermal control unit (AirTherm SMT, World Precision Instruments) to ensure a constant temperature of 37 °C during growth curve measurements. The plate stacker constantly read plates and one complete run through all plates required 30–42 min depending on the number of plates. The plate reader settings were as follows: 37 °C, 10 s of shaking at 282 cycles per min with a double orbital pattern before reading optical density at 600 nm. After approximately 48 h of growth, the plates were removed from the plate stacker and used for single-cell imaging.

### Storing glycerol stocks

After being used to inoculate flat-bottom plates for growth curves, the deep well cultures were sealed with a plastic film (Excel Scientific, Cat. #STR-SEAL-PLT) and transferred back to an anaerobic chamber using an airtight box. The BenchSmart 96-well pipetting robot was used along with a P1000 head and a 300-mL reservoir to transfer 353 μL of a sterile solution of 50% glycerol (Fisher, Cat. #G33-4) and 50 mM cysteine (Millipore, Cat. #243005-100GM) that had been pre-reduced inside the chamber for >48 h. After mixing the cultures with glycerol by pipetting up and down twice, the glycerol stocks were distributed in 80-μL aliquots into 96-well V-bottom plates covered temporarily with a sterile lid (Greiner, Cat. #656161) as copies of the final condensed library. Aliquoted library copies were sealed with a foil seal, the sterile lid was placed over the seal, and plates were transferred to a −80 °C freezer for long-term storage.

### Pooling strategy

Wells in the progenitor collection (1st round) and ordered collection (2nd round, quality check) were pooled according to the same plate-well strategy. A plate-well pooling strategy requires *N*+96 pools, where *N* is the number of 96-well plates. Pooling essentially followed the procedure described previously [30]. Individual wells were first pooled, either pooling the same well from all plates (e.g., A1 from progenitor collection plates 41–302) or pooling all wells from a single plate (e.g., A1–H12 from plate 41). In previous efforts [30], the first pool set (same well, different plates) was pooled further to create 8 and 12 row and column pools, respectively. In this work, the set of pools from the 96-well positions were sequenced directly, along with the 262 plate pools. As described previously [30], a single pool was made from every well of the progenitor collection extension (plates 41–302) for use as input to RB-TnSeq.

With the plate-well pooling strategy used here, the location of a barcode isolated *n* times in the collection will be narrowed down to *n*^2^ possible wells. For example, a barcode isolated at position G1 of plate 1 (P1-G1) and position H2 of plate 2 (P2-H2) of the progenitor collection will be sequenced in pools P1, P2, G1, and H2. The four possible locations that are consistent with these results are P1-G1, P1-H2, P2-G1, and P2-H2. Two of the possible locations in this example are the true locations, while the remaining two are artifacts of the pooling and decoding process.

When a barcode did not have a definite location, we used a probabilistic strategy to predict the likelihood of a particular configuration of wells, as described previously [30, 53]. Critically, this algorithm depends on systematic differences in the contribution of each well in the pool to the total number of sequencing reads [53]. While BarSeq is particularly effective compared to other methods at providing a quantitative and accurate estimate of the relative abundance of a barcode in the pool [30], plate-well pools carrying similar relative abundance of the barcode in question are poor in information and hence the resulting predictions are low in confidence. Here, we used a heuristic cutoff of 0.85 for considering a predicted configuration of locations to be high confidence. If the probability was <0.85, all possible wells from one plate were transferred to the condensed collection, and the true mutant was identified in the subsequent quality check sequencing run. With the plate-well pooling strategy used here, barcodes isolated *n* times with ambiguous locations resulted in *n* wells being transferred to the condensed collection, only one of which contained the correct mutant.

### Sequencing the progenitor collection

DNA from plate-well pools of the progenitor collection was isolated with a DNeasy 96 Blood &Tissue Kit (Qiagen, Cat. #69582) according to the manufacturer’s instructions. BarSeq was performed on individual pools with indexed primers (Table S1), as described previously [30], and sequenced on a MiSeq (Illumina, SY-410-1003) using MiSeq Reagent Kit v3 (150-cycle) (Illumina, MS-102-3001). DNA was isolated from the complete pool at the same time and used as input for an RB-TnSeq protocol, as detailed previously [30] (Table S1). The RB-TnSeq library was sequenced on a MiSeq using MiSeq Reagent Kit v3 (150-cycle).

### Decoding the progenitor collection

The process of locating barcodes within the progenitor collection was performed essentially as previously described for the initial 40-plate collection [30], with small modifications to account for the change in pooling strategy from row-column-plate to plate-well. Briefly, we used the BarSeq results from individual pools to locate barcodes within the collection. Barcodes with definite solutions (isolated once in the collection) were identified first, then statistics on the distribution of abundance of these barcodes were used to inform the likelihood of solutions for the location of barcodes without definite solutions (isolated more than once in the collection).

Simultaneously, we incorporated the RB-TnSeq data of the progenitor collection into the larger RB-TnSeq dataset from the initial pooled library. The higher depth of sequencing from RB-TnSeq on the progenitor collection allowed us to map more barcodes to the genome and provided higher sensitivity for the detection of multiple insertion sites associated with the same barcode. Once a barcode was located in the collection, a lookup table connecting barcodes to insertion sites was used to determine its utility as a mutant strain for the condensed collection.

The detailed algorithm for determining the mapping status of a barcode (e.g., single-insertion versus multiple/ambiguous insertion) can be found in previously published code [30]. Importantly, we only considered insertion locations in the genome for which the number of reads was >25% that of the most abundant insertion location for the same barcode. While the same barcode mapping to multiple locations in the genome in a pooled library could arise from multiple causes (such as the chance occurrence of the same barcode in multiple strains), an RB-TnSeq dataset from the progenitor collection alone indicated that the majority of barcodes that mapped to multiple sites were isolated only once, consistent with previous results [30]. If the insertions of these ambiguous barcodes were found in separate strains, this scenario would require the repeated sorting of two or more cells with the same barcode into the same well. Therefore, in this study we treated barcodes associated with more than one insertion site as multiple-insertion strains.

### Modeling assembly of the progenitor collection

To quantify barcode abundance in the initial pool, a 1-mL aliquot of the same initial pool as the one used for sorting the additional 262-plates was inoculated into BHIS and recovered overnight at 37 °C. Six 1-mL aliquots of this overnight culture were pelleted, DNA was extracted, and BarSeq was performed as above (see “Sequencing the progenitor collection”). This protocol is the same as the one used to generate the *t*_0_ samples that serve as controls for pooled fitness assays, and we expect that any *t*_0_ sequencing data will be useful for modeling collection assembly as long as it comes from the same initial pool as the culture used for sorting and was recovered for a similar period of time. The relative barcode abundances were averaged across these *t*_0_ samples and used as a probabilistic weight for random sampling during simulations. Before drawing from the pool, we filtered out barcodes in the *t*_0_ samples that were not associated with any insertion location (unmapped).

We started with a quantification of strain abundance in the initial pool used to sort the library using BarSeq. In our simulation, we used a Monte Carlo approach (repeated random sampling, weighted by relative abundance) [53] to simulate the isolation of barcodes from the initial pool and calculated the genome coverage (number of genes represented by ≥1 insertions) across a range of progenitor collection size. Barcodes were randomly drawn with replacement from an initial set defined by the initial pool, insertion sites were located, and genome coverage was determined. We required that an insertion be found between the first 5% and last 10% of a gene to consider that gene disrupted. We simulated a range of collection sizes (*b* total barcodes) and performed 250 simulations for each collection size. To account for assembly efficiency (*K*)*,* we scaled the collection size in the simulated coverage curves by *K*^−1^. To assess the impact of strain abundance bias, we simulated collection assembly from an initial pool in which the weights were set to be equal. To assess the impact of assembly efficiency, we set *K*=1.

The value of *K* was estimated by quantifying two parameters from the statistics of the 262-plate library: *bpw*, the number of barcode bins per well, and *f*_single_, the fraction of barcode bins associated with a single site. These parameters were chosen because they represent the filtering steps used in this work to determine whether a barcode was useful for inclusion in the final condensed collection. We expect that *K* can be estimated for any protocol, as long as the fraction of wells with a useful barcode is accurately quantified.

### Measurement of population growth metrics

Maximum growth rate was calculated as the largest slope of ln(OD) with respect to time (calculated from a linear regression of a sliding window of 5 time points) using custom Matlab (Mathworks, Natick, MA, USA) code.

### Single-cell imaging

Stationary-phase cells or cells from cryostocks were diluted 1:10 into 0.85X PBS and then taken from 96-well plates and placed on 1% agarose pads with 0.85X PBS to control for osmolality. Phase-contrast images were acquired with a Ti-E inverted microscope (Nikon Instruments) using a 100X (NA 1.40) oil immersion objective and a Neo 5.5 sCMOS camera (Andor Technology). Images were acquired using μManager v.1.4 [54]. High-throughput imaging was accomplished using SLIP, as described previously [37]. Including sample preparation and calibration, SLIP enables acquisition of 49 images per well of a 96-well plate in ~30 min. Since replicate growth curves appeared similar across the entire library (Additional file 4: Fig. S2), we imaged one replicate culture for each strain.

### Morphological analyses

The MATLAB image processing code *Morphometrics* [34] was used to segment cells from phase-contrast or fluorescence microscopy images. A local coordinate system was generated for each cell outline using a method adapted from *MicrobeTracker* [55]. Cell widths were calculated by averaging the distances between contour points perpendicular to the cell midline, excluding contour points within the poles and sites of septation. Cell length was calculated as the length of the midline from pole to pole. Cell surface area was estimated from the local meshing.

### Growth curve measurements from single colonies

To isolate single colonies, we aerobically struck glycerol stocks onto BHIS+1.5% agar plates and then transferred the plates to an anaerobic chamber and incubated them at 37 °C for 48 h. We performed all further steps in an anaerobic chamber. We inoculated single colonies into a pre-reduced and pre-blanked flat-bottom plate (Greiner Bio-One, Cat. #655180) with 200 μL of pre-reduced BHIS and into a 96-deep well plate (Greiner Bio-One, Cat. #780280) with 500 μL of pre-reduced BHIS, and incubated the cultures for 48 h. The flat-bottom plate was used to measure outgrowth from the colony and the deep well plate was incubated without shaking in a 37 °C incubator. Since BT0870 colonies were visible but much smaller than wild type, we combined 5–6 colonies of the BT0870 mutant into one culture to approximately normalize the inoculum density. Two microliters of deep well cultures was used to inoculate a pre-reduced and pre-blanked flat-bottom plate with 200 μL of pre-reduced BHIS. For measurements of both outgrowth from a colony and from the 48 h cultures, we applied an optical seal (Excel Scientific, Cat. #STR-SEAL-PLT) and measured OD_600_ with a Biotek Epoch plate reader with the following settings: temperature 37 °C, reading of OD_600_ every 5 min with continual orbital shaking (3 mm, 282 cycles per min) between reads. We subtracted well-specific blank values before plotting the growth curves and calculating maximum growth rate [56].

### PCR confirmation of double-insertion strains

We chose representative strains from the progenitor and ordered collections for confirmation of sequencing-based classification of either single-barcode double-insertion or double-barcode double-insertion strains. A common forward primer within the transposon was paired with an insertion-specific reverse primer, and a PCR check was performed to confirm the transposon-insertion site by amplifying across the transposon-insertion junction (Table S1). A sample from targeted wells in the collection was struck out on BHIS plates, eight individual colonies per strain were picked and grown up overnight, and the overnight cultures were used as input for colony PCR. If both insertion sites could be detected in most single colonies, the strain was considered as a confirmed multi-insertion strain.

## Supplementary Information

**Additional file 1: Figure S1.** The progenitor collection expands coverage of the *B. theta* genome. A) The genes covered by single insertions in the progenitor collection overlap to some degree with a previously published [23] ordered collection of *B. theta* VPI-5482 transposon mutants. The Venn diagram shows the number of genes that overlap between the two datasets (762) and the number that are unique to the progenitor collection (1,740) and the previously published collection (748). Information on insertion locations in the previous collection was extracted from published materials and reanalyzed using the same criteria for coverage as the collection in this work. B) The progenitor collection expands coverage of the *B. theta* genome. The number of genes represented by transposon mutant strains is shown for the progenitor collection (top) and the previously reported collection (bottom). As in (A), information on insertion location was extracted from the previous publication and reanalyzed. To allow for direct comparison between datasets, only genes on the chromosome of *B. theta* VPI-5482 were considered in this analysis (plasmid-encoded genes were excluded from analysis). C) The distribution of transposon insertions within open reading frames reflects the selection criteria for the condensed collection. The distribution of transposon-insertion locations is plotted as a function of relative position in open reading frames for the initial pool (top, black), progenitor collection (middle, green), and condensed collection (bottom, yellow). Transposon insertions that occur outside of open reading frames or that occur on the plasmid were excluded. The distribution  is essentially uniform in the initial pool (*~*100,000 insertions) and progenitor collection (*~*9,000 insertions), reflecting both the random nature of transposition and the random selection of strains in the sorting procedure. Insertions are missing from the first 5% and last 10% and biased toward the center of open reading frames in the condensed collection (>2,500 insertions), reflecting the selection criteria in the final re-arraying step.

**Additional file 2: Dataset S1.** The condensed collection.

**Additional file 3: Dataset S2.** Genes covered only in multi-insertion strains.

**Additional file 4: Figure S2.** Technical replicates of growth measurements from the condensed collection consistently highlight the same set of mutants with growth defects. A) A technical replicate of growth curves of the condensed collection led to identification of the same set of mutants with growth defects. Similar growth defects were observed for BT2397 (*tnr3*, blue), BT0870 (*spt*, red), and BT1439 (SusD-like, yellow) mutants as in the first replicate (Fig. 4B). The majority of strains grew similarly to wild-type controls (black, with shaded dark gray region representing 3 standard deviations). B) Maximum growth rate and maximum OD_600_ extracted from the technical replicate growth curves in (A) highlight the growth defects of BT2397 (*tnr3,* blue), BT0870 (*spt,* red), and BT1439 (SusD-like, yellow) mutants. C) Maximum growth rate was consistent between technical replicates of the growth curves. Pearson’s correlation coefficient *r*=0.63 (3 outliers excluded). Black circles are wild-type controls, which exhibited similar spread as the condensed collection. D) Maximum OD_600_ was reasonably consistent between technical replicates of the growth curves. Growth curves with OD_600_ much higher than wild type were generally not reproducible between replicates. Pearson’s correlation coefficient *r*=0.73 (3 outliers excluded). Black circles are wild-type controls.

**Additional file 5: Figure S3.** The BT0870 (*spt*) mutant exhibits growth defects during outgrowth from a colony. Growth curves are of wild-type *B. theta* VPI-5482 (wild type, black) and BT0870 (*spt*, red) cultures inoculated directly from colonies into liquid BHIS. BT0870 (*spt*) displayed qualitatively similar growth curves to cultures inoculated from a liquid passage after colony growth (Fig. 4D). Shaded regions represent 1 standard deviation for *n*=6 biological replicates.

**Additional file 6: Figure S4.** The elongated cell phenotype of the BT2937 (*tnr3*) mutant is specific to passaging through liquid. A) Independent strains with insertions in BT2397 (*tnr3*) exhibited elongated cells, including the BT2397 (*tnr3*) cryostock from the condensed collection and two independent strains from the progenitor collection (one was the single-insertion BT2397 (*tnr3*) mutant that was propagated for the condensed collection and the other has a barcode associated with insertions in BT2397 and BT2343). Cells were spotted directly onto agarose pads after dilution from the cryostock and imaged aerobically without growth. Scale bar: 20 μm. B) Cultures inoculated by sorting a single cell into liquid BHIS and passaged twice exhibited an increased fraction of elongated cells, while cultures inoculated from a colony and passaged twice in liquid BHIS (blue) before imaging displayed uniform, approximately wild-type shapes. Sorting was performed with either a gate to select for small, approximately wild-type shaped cells (green) or a gate to select for larger cells (purple), and cells were passaged in liquid BHIS twice before imaging; in both cases, a substantial fraction of cultures contained >2% of cells with area >3 μm^2^, unlike cultures inoculated from a colony. Representative images are shown in the inset. Scale bar: 10 μm. *n*>300 cells were segmented per culture, from 64, 83, and 63 cultures for passaging through a colony, inoculated with a small cell, or inoculated with a large cell, respectively.

**Additional file 7: Table S1.** Strains and oligos used in this study.

**Additional file 8: Table S2.** Program settings for re-arraying.

**Additional file 9: Table S3.** Program settings for the inoculation of growth curves.

## Data Availability

The data used to generate the figures of this paper are available at Dryad [57] and a repository of the code is maintained at Bitbucket [58]. Sequencing data have been deposited at NCBI SRA with BioProject PRJNA888137 [59]. Strains and copies of the ordered collection are available from the corresponding authors (kchuang@stanford.edu, ashiver@stanford.edu ) with a completed Material Transfer Agreement.
