# Supplementary figures and images for: Construction and characterization of a genome-scale ordered mutant collection of Bacteroides thetaiotaomicron
Source: BMC Biol. 2022 Dec 17;20:285. doi: 10.1186/s12915-022-01481-2 (PMC9758874; doi:10.1186/s12915-022-01481-2)

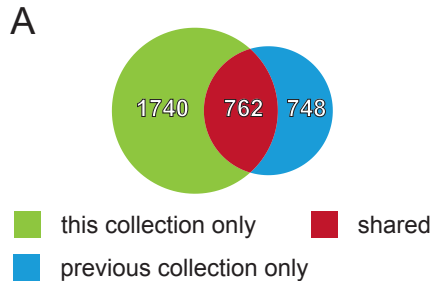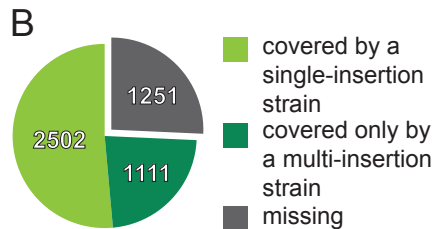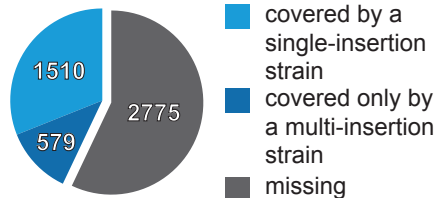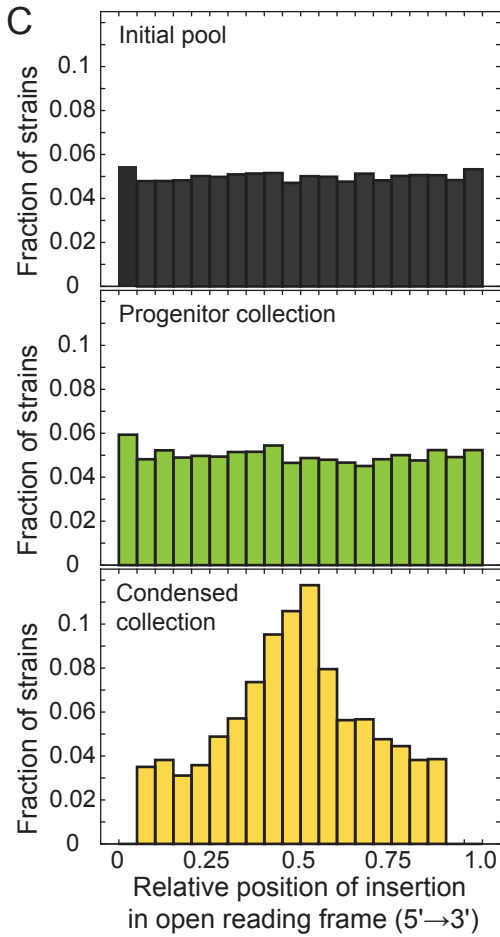

Supplement: Supplementary file 1 — Additional file 1: Figure S1. The progenitor collection expands coverage of the B. theta genome. A) The genes covered by single insertions in the progenitor collection overlap to some degree with a previously published [23] ordered collection of B. theta VPI-5482 transposon mutants. The Venn diagram shows the number of genes that overlap between the two datasets (762) and the number that are unique to the progenitor collection (1,740) and the previously published collection (748). Information on insertion locations in the previous collection was extracted from published materials and reanalyzed using the same criteria for coverage as the collection in this work. B) The progenitor collection expands coverage of the B. theta genome. The number of genes represented by transposon mutant strains is shown for the progenitor collection (top) and the previously reported collection (bottom). As in (A), information on insertion location was extracted from the previous publication and reanalyzed. To allow for direct comparison between datasets, only genes on the chromosome of B. theta VPI-5482 were considered in this analysis (plasmid-encoded genes were excluded from analysis). C) The distribution of transposon insertions within open reading frames reflects the selection criteria for the condensed collection. The distribution of transposon-insertion locations is plotted as a function of relative position in open reading frames for the initial pool (top, black), progenitor collection (middle, green), and condensed collection (bottom, yellow). Transposon insertions that occur outside of open reading frames or that occur on the plasmid were excluded. The distribution is essentially uniform in the initial pool (~100,000 insertions) and progenitor collection (~9,000 insertions), reflecting both the random nature of transposition and the random selection of strains in the sorting procedure. Insertions are missing from the first 5% and last 10% and biased toward the center of open re [file 12915_2022_1481_MOESM1_ESM.pdf]

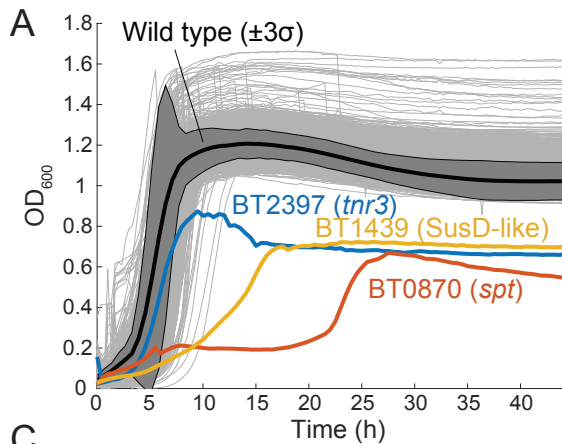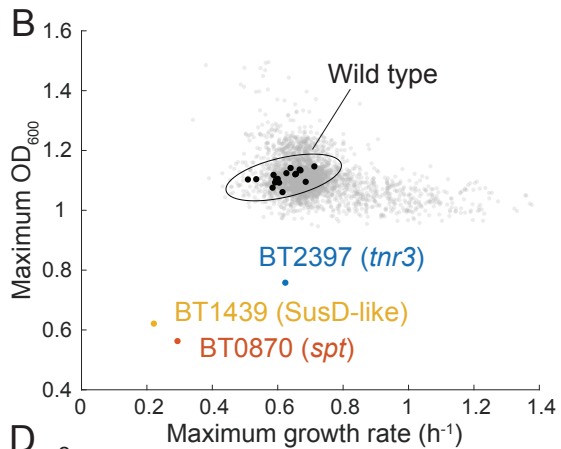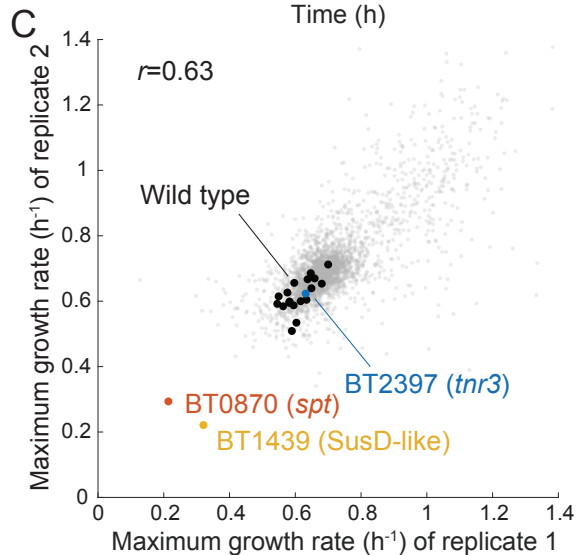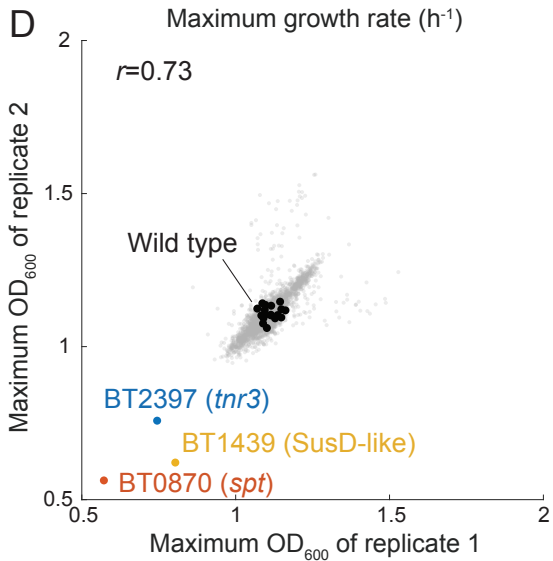

Supplement: Supplementary file 4 — Additional file 4: Figure S2. Technical replicates of growth measurements from the condensed collection consistently highlight the same set of mutants with growth defects. A) A technical replicate of growth curves of the condensed collection led to identification of the same set of mutants with growth defects. Similar growth defects were observed for BT2397 (tnr3, blue), BT0870 (spt, red), and BT1439 (SusD-like, yellow) mutants as in the first replicate (Fig. 4B). The majority of strains grew similarly to wild-type controls (black, with shaded dark gray region representing 3 standard deviations). B) Maximum growth rate and maximum OD600 extracted from the technical replicate growth curves in (A) highlight the growth defects of BT2397 (tnr3, blue), BT0870 (spt, red), and BT1439 (SusD-like, yellow) mutants. C) Maximum growth rate was consistent between technical replicates of the growth curves. Pearson’s correlation coefficient r=0.63 (3 outliers excluded). Black circles are wild-type controls, which exhibited similar spread as the condensed collection. D) Maximum OD600 was reasonably consistent between technical replicates of the growth curves. Growth curves with OD600 much higher than wild type were generally not reproducible between replicates. Pearson’s correlation coefficient r=0.73 (3 outliers excluded). Black circles are wild-type controls. [file 12915_2022_1481_MOESM4_ESM.pdf]

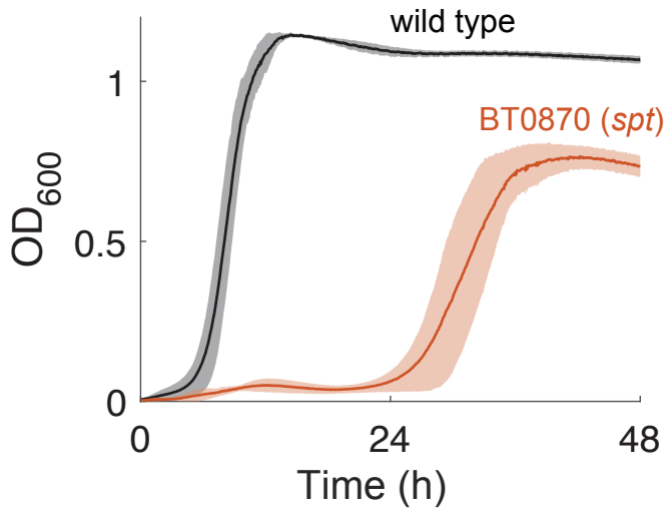

Supplement: Supplementary file 5 — Additional file 5: Figure S3. The BT0870 (spt) mutant exhibits growth defects during outgrowth from a colony. Growth curves are of wild-type B. theta VPI-5482 (wild type, black) and BT0870 (spt, red) cultures inoculated directly from colonies into liquid BHIS. BT0870 (spt) displayed qualitatively similar growth curves to cultures inoculated from a liquid passage after colony growth (Fig. 4D). Shaded regions represent 1 standard deviation for n=6 biological replicates. [file 12915_2022_1481_MOESM5_ESM.pdf]
