# Supplementary material for: Construction and characterization of a genome-scale ordered mutant collection of Bacteroides thetaiotaomicron
Source: BMC Biol. 2022 Dec 17;20:285. doi: 10.1186/s12915-022-01481-2 (PMC9758874; doi:10.1186/s12915-022-01481-2)
Supplement: Supplementary file 6 — Additional file 6: Figure S4. The elongated cell phenotype of the BT2937 (tnr3) mutant is specific to passaging through liquid. A) Independent strains with insertions in BT2397 (tnr3) exhibited elongated cells, including the BT2397 (tnr3) cryostock from the condensed collection and two independent strains from the progenitor collection (one was the single-insertion BT2397 (tnr3) mutant that was propagated for the condensed collection and the other has a barcode associated with insertions in BT2397 and BT2343). Cells were spotted directly onto agarose pads after dilution from the cryostock and imaged aerobically without growth. Scale bar: 20 μm. B) Cultures inoculated by sorting a single cell into liquid BHIS and passaged twice exhibited an increased fraction of elongated cells, while cultures inoculated from a colony and passaged twice in liquid BHIS (blue) before imaging displayed uniform, approximately wild-type shapes. Sorting was performed with either a gate to select for small, approximately wild-type shaped cells (green) or a gate to select for larger cells (purple), and cells were passaged in liquid BHIS twice before imaging; in both cases, a substantial fraction of cultures contained >2% of cells with area >3 μm2, unlike cultures inoculated from a colony. Representative images are shown in the inset. Scale bar: 10 μm. n>300 cells were segmented per culture, from 64, 83, and 63 cultures for passaging through a colony, inoculated with a small cell, or inoculated with a large cell, respectively. [file 12915_2022_1481_MOESM6_ESM.pdf]

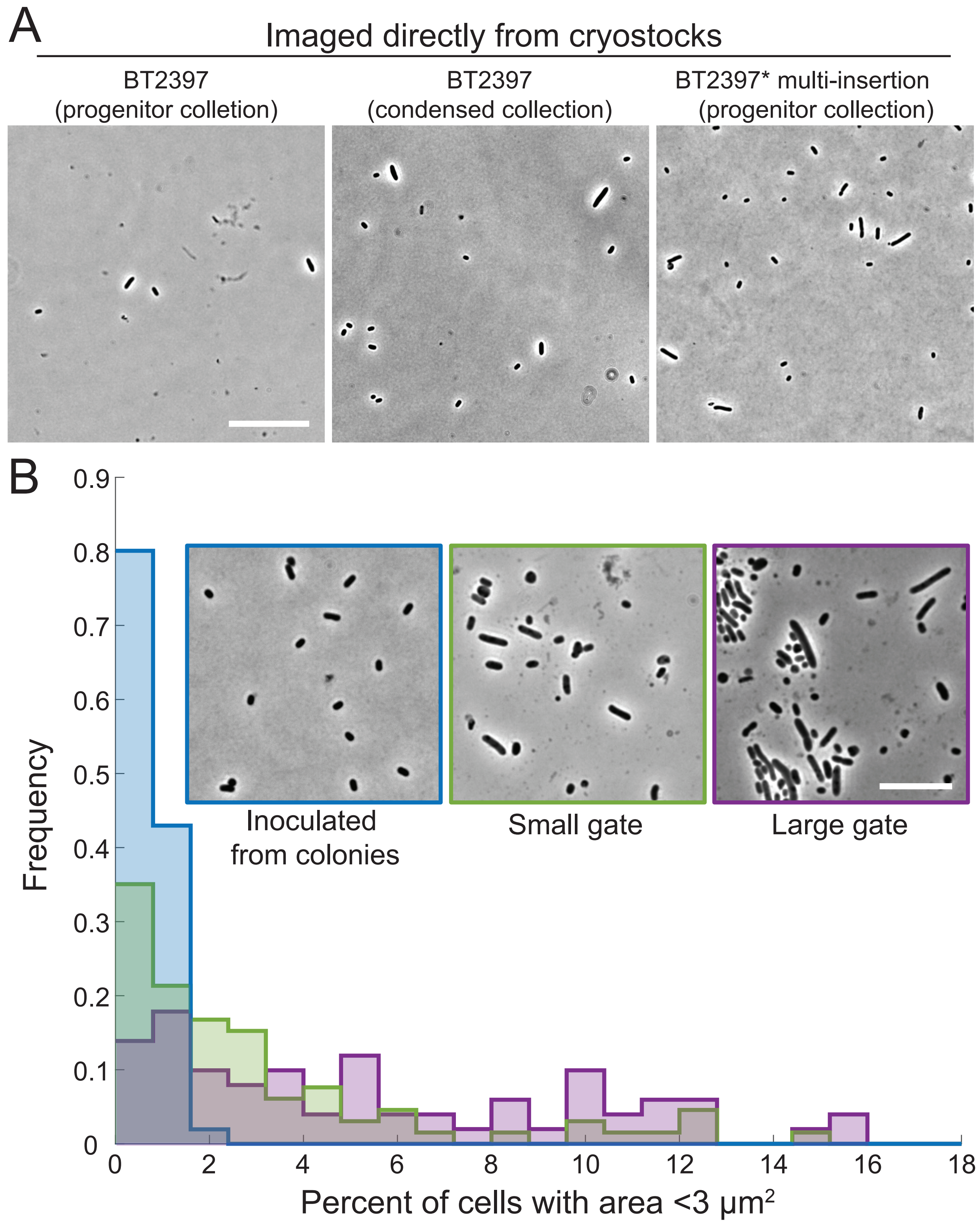

A
Imaged directly from cryostocks
BT2397 (progenitor colletion)
BT2397 (condensed collection)
BT2397* multi-insertion (progenitor collection)
B
Frequency
0.9
0.8
0.7
0.6
0.5
0.4
0.3
0.2
0.1
0
Inoculated from colonies
Small gate
Large gate
0
2
4
6
8
10
12
14
16
18
Percent of cells with area <3 μm²
