# Supplementary material for: Construction and characterization of a genome-scale ordered mutant collection of Bacteroides thetaiotaomicron
Source: BMC Biol. 2022 Dec 17;20:285. doi: 10.1186/s12915-022-01481-2 (PMC9758874; doi:10.1186/s12915-022-01481-2)
Supplement: Supplementary file 7 — Additional file 7: Table S1. Strains and oligos used in this study. [file 12915_2022_1481_MOESM7_ESM.docx]

**Table S1: Strains and oligos used in this study.**

| **Strains used in this study** | | | | | | | | | | |
| --- | --- | --- | --- | --- | --- | --- | --- | --- | --- | --- |
| **Strain name** | **Gene disrupted** | **Progenitor collection location** | **Condensed collection location** | **Insertion location** | | **Insertion orientation** | **Insertion scaffold** | | **Source** | |
| *B. thetaiotaomicron* VPI-4582 | - | - | - | - | | - | - | | Laboratory of Justin Sonnenburg | |
| BT0870 | BT0870 | P7-G9 | P63-C03 | 1070571 | | - | Chr | | This work | |
| BT1439 | BT1439 | P28-E05 | P288-B02 | 1770536 | | - | Chr | | This work | |
| BT2397 | BT2379 | P38-G10 | P5-E07 | 2992939 | | + | Chr | | This work | |
| BT2397* | BT2379; BT2343; BT4114 | P55-F10 | - | 2992939; 2923983;5387641 | | +; +; + | Chr; Chr; Chr | | This work | |
| **Oligos used to confirm insertion locations** | | | | | | | | | | |
| **Oligo name** | **Notes** | **Oligo sequence** | | | | | | | **Source** | |
| Erm-F | Common forward primer in erm transposon | GCAGGTAATACGACTCACTATAG | | | | | | | [30] | |
| BT0870-1070571(-) | *spt* check reverse primer | gcgataaaaccgccgatagagg | | | | | | | This work | |
| BT2397-2992939(+) | *tnr3* check reverse primer | ctgtccgtacttccataccacttctc | | | | | | | This work | |
| PC-103-A08-S1 | single-barcode double-insertion P103-A08 site-1 | GAGGGGTTTGACAGGGCTAC | | | | | | | This work | |
| PC-103-A08-S1 | single-barcode double-insertion P103-A11 site-1 | GGCACCGACGGATATCACTT | | | | | | | This work | |
| PC-103-B03-S1 | single-barcode double-insertion P103-B03 site-1 | TCCAGAGCAGCAAACGTCAT | | | | | | | This work | |
| PC-103-B09-S1 | single-barcode double-insertion P103-B09 site-1 | CCAGTGCCGTCTTTTCCTCT | | | | | | | This work | |
| PC-103-C02-S1 | single-barcode double-insertion P103-C02 site-1 | AGGGTGCAGCTTTTGAAGGT | | | | | | | This work | |
| PC-103-C11-S1 | single-barcode double-insertion P103-C11 site-1 | ACGCACGGCTAAATCTGGAA | | | | | | | This work | |
| PC-103-D12-S1 | single-barcode double-insertion P103-D12 site-1 | GACCTCGACGACTTCTTCCC | | | | | | | This work | |
| PC-103-E08-S1 | single-barcode double-insertion P103-E08 site-1 | CTTGCAGGGAAGTGAGCAGA | | | | | | | This work | |
| PC-103-E09-S1 | single-barcode double-insertion P103-E09 site-1 | GCAGACGCCCTCTACTTTGT | | | | | | | This work | |
| PC-103-F01-S1 | Single-barcode double-insertion P103-F01 site-1 | GAGTTCTGTTTGCTGTGGCG | | | | | | | This work | |
| PC-103-G09-S1 | single-barcode double-insertion P103-G09 site-1 | GCATGGCGGCTATCATAAGC | | | | | | | This work | |
| PC-103-G12-S1 | single-barcode double-insertion P103-G12 site-1 | ACCAGCAACATAAGGGTGCA | | | | | | | This work | |
| PC-103-A08-S2 | single-barcode double-insertion P103-A08 site-2 | TGGGGCGGGCATAGAAAAAT | | | | | | | This work | |
| PC-103-A08-S2 | single-barcode double-insertion P103-A11 site-2 | TCTCTACAGATTCGGCGGGA | | | | | | | This work | |
| PC-103-B03-S2 | single-barcode double-insertion P103-B03 site-2 | GCGCGTTCGGTAAAGCATAA | | | | | | | This work | |
| PC-103-B09-S2 | single-barcode double-insertion P103-B09 site-2 | GGTTAATGTCGGCAATCGGC | | | | | | | This work | |
| PC-103-C02-S2 | single-barcode double-insertion P103-C02 site-2 | GGATTGTCAGAACTGCCCGA | | | | | | | This work | |
| PC-103-C11-S2 | single-barcode double-insertion P103-C11 site-2 | TCAAACCAGCGATGTCCCTC | | | | | | | This work | |
| PC-103-D12-S2 | single-barcode double-insertion P103-D12 site-2 | GGGCACGAAATACCAGTGGA | | | | | | | This work | |
| PC-103-E08-S2 | single-barcode double-insertion P103-E08 site-2 | GCCCGCGGTACAGAATATGA | | | | | | | This work | |
| PC-103-E09-S2 | single-barcode double-insertion P103-E09 site-2 | GCCCCTGCATTCCCTTTACT | | | | | | | This work | |
| PC-103-F01-S2 | single-barcode double-insertion P103-F01 site-2 | AAATGGAATCCACTGCCGGT | | | | | | | This work | |
| PC-103-G09-S2 | single-barcode double-insertion P103-G09 site-2 | ACTTCATGGGCGAACCTCAG | | | | | | | This work | |
| PC-103-G12-S2 | single-barcode double-insertion P103-G12 site-2 | CTGCCGCATAACCATTGACG | | | | | | | This work | |
| CC-13-D04-S1 | double-barcode double-insertion P13-D04 site-1 | CGGAGTTGACTATTGCCGGT | | | | | | | This work | |
| CC-14-A03-S1 | double-barcode double-insertion P14-A03 site-1 | GTTGTCCGGACGGTATGTGA | | | | | | | This work | |
| CC-14-E02-S1 | double-barcode double-insertion P14-E02 site-1 | CGAGAACTGGCATGGACTGT | | | | | | | This work | |
| CC-15-A08-S1 | double-barcode double-insertion P15-A08 site-1 | CTCATCCGCCTGCTCGTTAT | | | | | | | This work | |
| CC-15-D09-S1 | double-barcode double-insertion P15-D09 site-1 | GTTTGTAGCGGCAATAGCGG | | | | | | | This work | |
| CC-15-G10-S1 | double-barcode double-insertion P15-G10 site-1 | GTCGGCTTTCCAGCTACCAT | | | | | | | This work | |
| CC-16-C08-S1 | double-barcode double-insertion P16-C08 site-1 | GCTCCACGCTATGATGACGA | | | | | | | This work | |
| CC-17-F08-S1 | double-barcode double-insertion P17-F08 site-1 | ACAGAGGAATGGAACCGCAG | | | | | | | This work | |
| CC-19-C02-S1 | double-barcode double-insertion P19-A02 site-1 | TGCTTGCGTGGAATCCTGAT | | | | | | | This work | |
| CC-19-F08-S1 | double-barcode double-insertion P19-F08 site-1 | AGGCAGTAGCAACACAAGCA | | | | | | | This work | |
| CC-13-D04-S2 | double-barcode double-insertion P13-D04 site-2 | CGCATCCATTGACTCGCAAG | | | | | | | This work | |
| CC-14-A03-S2 | double-barcode double-insertion P14-A03 site-2 | TGCGATCGTCGGTTTCTCAA | | | | | | | This work | |
| CC-14-E02-S2 | double-barcode double-insertion P14-E02 site-2 | AGAACTTCCGCAGAATGGCA | | | | | | | This work | |
| CC-15-A08-S2 | double-barcode double-insertion P15-A08 site-2 | ATCTCACAGGTACGCGCAAT | | | | | | | This work | |
| CC-15-D09-S2 | double-barcode double-insertion P15-D09 site-2 | GATGATGCGTCGTTTGCTCC | | | | | | | This work | |
| CC-15-G10-S2 | double-barcode double-insertion P15-G10 site-2 | ATCCCATACCACTCCACGGA | | | | | | | This work | |
| CC-16-C08-S2 | double-barcode double-insertion P16-C08 site-2 | ATCCCCACCCAGGTCCTATC | | | | | | | This work | |
| CC-17-F08-S2 | double-barcode double-insertion P17-F08 site-2 | TACGAACCAGCCATATCCGC | | | | | | | This work | |
| CC-19-C02-S2 | double-barcode double-insertion P19-A02 site-2 | TTCCCTTTCCATCGCCTAGC | | | | | | | This work | |
| CC-19-F08-S2 | double-barcode double-insertion P19-F08 site-2 | TCGCTTCCTGATACGTACGC | | | | | | | This work | |
| **Oligos used for BarSeq** | | | | | | | | | | |
| **Oligo name** | **Notes** | **Sequence** | | | **Index name** | | | **Index** | | **Source** |
| P5-std-BarSeq-i503 | Forward primer for BarSeq, index i503 | AATGATACGGCGACCACCGAGATCTACAC **CCTATCCT** ACACTCTTTCCCTACACGACGCTCTTCCGATCT NN  GTCGACCTGCAGCGTACG | | | i503 | | | CCTATCCT | | This work |
| P5-std-BarSeq-i504 | Forward primer for BarSeq, index i504 | AATGATACGGCGACCACCGAGATCTACAC **GGCTCTGA** ACACTCTTTCCCTACACGACGCTCTTCCGATCT NN  GTCGACCTGCAGCGTACG | | | i504 | | | GGCTCTGA | | This work |
| P5-std-BarSeq-i506 | Forward primer for BarSeq, index i505 | AATGATACGGCGACCACCGAGATCTACAC **TAATCTTA** ACACTCTTTCCCTACACGACGCTCTTCCGATCT NN  GTCGACCTGCAGCGTACG | | | i506 | | | TAATCTTA | | This work |
| P5-std-BarSeq-i507 | Forward primer for BarSeq, index i506 | AATGATACGGCGACCACCGAGATCTACAC **CAGGACGT** ACACTCTTTCCCTACACGACGCTCTTCCGATCT NN  GTCGACCTGCAGCGTACG | | | i507 | | | CAGGACGT | | This work |
| Barseq_P2_IT 001 | Reverse primer for Barseq, index IT001 | CAAGCAGAAGACGGCATACGAGATCGTGATGTGACTGGAGTTCAGACGTGTGCTCTTCCGATCTGATGTCCACGAGGTCTCT | | | IT001 | | | ATCACG | | [24] |
| Barseq_P2_IT 002 | Reverse primer for Barseq, index IT002 | CAAGCAGAAGACGGCATACGAGATACATCGGTGACTGGAGTTCAGACGTGTGCTCTTCCGATCTGATGTCCACGAGGTCTCT | | | IT002 | | | CGATGT | | [24] |
| Barseq_P2_IT 003 | Reverse primer for Barseq, index IT003 | CAAGCAGAAGACGGCATACGAGATGCCTAAGTGACTGGAGTTCAGACGTGTGCTCTTCCGATCTGATGTCCACGAGGTCTCT | | | IT003 | | | TTAGGC | | [24] |
| Barseq_P2_IT 004 | Reverse primer for Barseq, index IT004 | CAAGCAGAAGACGGCATACGAGATTGGTCAGTGACTGGAGTTCAGACGTGTGCTCTTCCGATCTGATGTCCACGAGGTCTCT | | | IT004 | | | TGACCA | | [24] |
| Barseq_P2_IT 005 | Reverse primer for Barseq, index IT005 | CAAGCAGAAGACGGCATACGAGATCACTGTGTGACTGGAGTTCAGACGTGTGCTCTTCCGATCTGATGTCCACGAGGTCTCT | | | IT005 | | | ACAGTG | | [24] |
| Barseq_P2_IT 006 | Reverse primer for Barseq, index IT006 | CAAGCAGAAGACGGCATACGAGATATTGGCGTGACTGGAGTTCAGACGTGTGCTCTTCCGATCTGATGTCCACGAGGTCTCT | | | IT006 | | | GCCAAT | | [24] |
| Barseq_P2_IT 007 | Reverse primer for Barseq, index IT007 | CAAGCAGAAGACGGCATACGAGATGATCTGGTGACTGGAGTTCAGACGTGTGCTCTTCCGATCTGATGTCCACGAGGTCTCT | | | IT007 | | | CAGATC | | [24] |
| Barseq_P2_IT 008 | Reverse primer for Barseq, index IT008 | CAAGCAGAAGACGGCATACGAGATTCAAGTGTGACTGGAGTTCAGACGTGTGCTCTTCCGATCTGATGTCCACGAGGTCTCT | | | IT008 | | | ACTTGA | | [24] |
| Barseq_P2_IT 009 | Reverse primer for Barseq, index IT009 | CAAGCAGAAGACGGCATACGAGATCTGATCGTGACTGGAGTTCAGACGTGTGCTCTTCCGATCTGATGTCCACGAGGTCTCT | | | IT009 | | | GATCAG | | [24] |
| Barseq_P2_IT 010 | Reverse primer for Barseq, index IT010 | CAAGCAGAAGACGGCATACGAGATAAGCTAGTGACTGGAGTTCAGACGTGTGCTCTTCCGATCTGATGTCCACGAGGTCTCT | | | IT010 | | | TAGCTT | | [24] |
| Barseq_P2_IT 011 | Reverse primer for Barseq, index IT011 | CAAGCAGAAGACGGCATACGAGATGTAGCCGTGACTGGAGTTCAGACGTGTGCTCTTCCGATCTGATGTCCACGAGGTCTCT | | | IT011 | | | GGCTAC | | [24] |
| Barseq_P2_IT 012 | Reverse primer for Barseq, index IT012 | CAAGCAGAAGACGGCATACGAGATTACAAGGTGACTGGAGTTCAGACGTGTGCTCTTCCGATCTGATGTCCACGAGGTCTCT | | | IT012 | | | CTTGTA | | [24] |
| Barseq_P2_IT 013 | Reverse primer for Barseq, index IT013 | CAAGCAGAAGACGGCATACGAGATTTGACTGTGACTGGAGTTCAGACGTGTGCTCTTCCGATCTGATGTCCACGAGGTCTCT | | | IT013 | | | AGTCAA | | [24] |
| Barseq_P2_IT 014 | Reverse primer for Barseq, index IT014 | CAAGCAGAAGACGGCATACGAGATGGAACTGTGACTGGAGTTCAGACGTGTGCTCTTCCGATCTGATGTCCACGAGGTCTCT | | | IT014 | | | AGTTCC | | [24] |
| Barseq_P2_IT 015 | Reverse primer for Barseq, index IT015 | CAAGCAGAAGACGGCATACGAGATTGACATGTGACTGGAGTTCAGACGTGTGCTCTTCCGATCTGATGTCCACGAGGTCTCT | | | IT015 | | | ATGTCA | | [24] |
| Barseq_P2_IT 016 | Reverse primer for Barseq, index IT016 | CAAGCAGAAGACGGCATACGAGATGGACGGGTGACTGGAGTTCAGACGTGTGCTCTTCCGATCTGATGTCCACGAGGTCTCT | | | IT016 | | | CCGTCC | | [24] |
| Barseq_P2_IT 017 | Reverse primer for Barseq, index IT017 | CAAGCAGAAGACGGCATACGAGATCTCTACGTGACTGGAGTTCAGACGTGTGCTCTTCCGATCTGATGTCCACGAGGTCTCT | | | IT017 | | | GTAGAG | | [24] |
| Barseq_P2_IT 018 | Reverse primer for Barseq, index IT018 | CAAGCAGAAGACGGCATACGAGATGCGGACGTGACTGGAGTTCAGACGTGTGCTCTTCCGATCTGATGTCCACGAGGTCTCT | | | IT018 | | | GTCCGC | | [24] |
| Barseq_P2_IT 019 | Reverse primer for Barseq, index IT019 | CAAGCAGAAGACGGCATACGAGATTTTCACGTGACTGGAGTTCAGACGTGTGCTCTTCCGATCTGATGTCCACGAGGTCTCT | | | IT019 | | | GTGAAA | | [24] |
| Barseq_P2_IT 020 | Reverse primer for Barseq, index IT020 | CAAGCAGAAGACGGCATACGAGATGGCCACGTGACTGGAGTTCAGACGTGTGCTCTTCCGATCTGATGTCCACGAGGTCTCT | | | IT020 | | | GTGGCC | | [24] |
| Barseq_P2_IT 021 | Reverse primer for Barseq, index IT021 | CAAGCAGAAGACGGCATACGAGATCGAAACGTGACTGGAGTTCAGACGTGTGCTCTTCCGATCTGATGTCCACGAGGTCTCT | | | IT021 | | | GTTTCG | | [24] |
| Barseq_P2_IT 022 | Reverse primer for Barseq, index IT022 | CAAGCAGAAGACGGCATACGAGATCGTACGGTGACTGGAGTTCAGACGTGTGCTCTTCCGATCTGATGTCCACGAGGTCTCT | | | IT022 | | | CGTACG | | [24] |
| Barseq_P2_IT 023 | Reverse primer for Barseq, index IT023 | CAAGCAGAAGACGGCATACGAGATCCACTCGTGACTGGAGTTCAGACGTGTGCTCTTCCGATCTGATGTCCACGAGGTCTCT | | | IT023 | | | GAGTGG | | [24] |
| Barseq_P2_IT 024 | Reverse primer for Barseq, index IT024 | CAAGCAGAAGACGGCATACGAGATGCTACCGTGACTGGAGTTCAGACGTGTGCTCTTCCGATCTGATGTCCACGAGGTCTCT | | | IT024 | | | GGTAGC | | [24] |
| Barseq_P2_IT 025 | Reverse primer for Barseq, index IT025 | CAAGCAGAAGACGGCATACGAGATATCAGTGTGACTGGAGTTCAGACGTGTGCTCTTCCGATCTGATGTCCACGAGGTCTCT | | | IT025 | | | ACTGAT | | [24] |
| Barseq_P2_IT 026 | Reverse primer for Barseq, index IT026 | CAAGCAGAAGACGGCATACGAGATGCTCATGTGACTGGAGTTCAGACGTGTGCTCTTCCGATCTGATGTCCACGAGGTCTCT | | | IT026 | | | ATGAGC | | [24] |
| Barseq_P2_IT 027 | Reverse primer for Barseq, index IT027 | CAAGCAGAAGACGGCATACGAGATAGGAATGTGACTGGAGTTCAGACGTGTGCTCTTCCGATCTGATGTCCACGAGGTCTCT | | | IT027 | | | ATTCCT | | [24] |
| Barseq_P2_IT 028 | Reverse primer for Barseq, index IT028 | CAAGCAGAAGACGGCATACGAGATCTTTTGGTGACTGGAGTTCAGACGTGTGCTCTTCCGATCTGATGTCCACGAGGTCTCT | | | IT028 | | | CAAAAG | | [24] |
| Barseq_P2_IT 029 | Reverse primer for Barseq, index IT029 | CAAGCAGAAGACGGCATACGAGATTAGTTGGTGACTGGAGTTCAGACGTGTGCTCTTCCGATCTGATGTCCACGAGGTCTCT | | | IT029 | | | CAACTA | | [24] |
| Barseq_P2_IT 030 | Reverse primer for Barseq, index IT030 | CAAGCAGAAGACGGCATACGAGATCCGGTGGTGACTGGAGTTCAGACGTGTGCTCTTCCGATCTGATGTCCACGAGGTCTCT | | | IT030 | | | CACCGG | | [24] |
| Barseq_P2_IT 031 | Reverse primer for Barseq, index IT031 | CAAGCAGAAGACGGCATACGAGATATCGTGGTGACTGGAGTTCAGACGTGTGCTCTTCCGATCTGATGTCCACGAGGTCTCT | | | IT031 | | | CACGAT | | [24] |
| Barseq_P2_IT 032 | Reverse primer for Barseq, index IT032 | CAAGCAGAAGACGGCATACGAGATTGAGTGGTGACTGGAGTTCAGACGTGTGCTCTTCCGATCTGATGTCCACGAGGTCTCT | | | IT032 | | | CACTCA | | [24] |
| Barseq_P2_IT 033 | Reverse primer for Barseq, index IT033 | CAAGCAGAAGACGGCATACGAGATCGCCTGGTGACTGGAGTTCAGACGTGTGCTCTTCCGATCTGATGTCCACGAGGTCTCT | | | IT033 | | | CAGGCG | | [24] |
| Barseq_P2_IT 034 | Reverse primer for Barseq, index IT034 | CAAGCAGAAGACGGCATACGAGATGCCATGGTGACTGGAGTTCAGACGTGTGCTCTTCCGATCTGATGTCCACGAGGTCTCT | | | IT034 | | | CATGGC | | [24] |
| Barseq_P2_IT 035 | Reverse primer for Barseq, index IT035 | CAAGCAGAAGACGGCATACGAGATAAAATGGTGACTGGAGTTCAGACGTGTGCTCTTCCGATCTGATGTCCACGAGGTCTCT | | | IT035 | | | CATTTT | | [24] |
| Barseq_P2_IT 036 | Reverse primer for Barseq, index IT036 | CAAGCAGAAGACGGCATACGAGATTGTTGGGTGACTGGAGTTCAGACGTGTGCTCTTCCGATCTGATGTCCACGAGGTCTCT | | | IT036 | | | CCAACA | | [24] |
| Barseq_P2_IT 037 | Reverse primer for Barseq, index IT037 | CAAGCAGAAGACGGCATACGAGATATTCCGGTGACTGGAGTTCAGACGTGTGCTCTTCCGATCTGATGTCCACGAGGTCTCT | | | IT037 | | | CGGAAT | | [24] |
| Barseq_P2_IT 038 | Reverse primer for Barseq, index IT038 | CAAGCAGAAGACGGCATACGAGATAGCTAGGTGACTGGAGTTCAGACGTGTGCTCTTCCGATCTGATGTCCACGAGGTCTCT | | | IT038 | | | CTAGCT | | [24] |
| Barseq_P2_IT 039 | Reverse primer for Barseq, index IT039 | CAAGCAGAAGACGGCATACGAGATGTATAGGTGACTGGAGTTCAGACGTGTGCTCTTCCGATCTGATGTCCACGAGGTCTCT | | | IT039 | | | CTATAC | | [24] |
| Barseq_P2_IT 040 | Reverse primer for Barseq, index IT040 | CAAGCAGAAGACGGCATACGAGATTCTGAGGTGACTGGAGTTCAGACGTGTGCTCTTCCGATCTGATGTCCACGAGGTCTCT | | | IT040 | | | CTCAGA | | [24] |
| Barseq_P2_IT 041 | Reverse primer for Barseq, index IT041 | CAAGCAGAAGACGGCATACGAGATGTCGTCGTGACTGGAGTTCAGACGTGTGCTCTTCCGATCTGATGTCCACGAGGTCTCT | | | IT041 | | | GACGAC | | [24] |
| Barseq_P2_IT 042 | Reverse primer for Barseq, index IT042 | CAAGCAGAAGACGGCATACGAGATCGATTAGTGACTGGAGTTCAGACGTGTGCTCTTCCGATCTGATGTCCACGAGGTCTCT | | | IT042 | | | TAATCG | | [24] |
| Barseq_P2_IT 043 | Reverse primer for Barseq, index IT043 | CAAGCAGAAGACGGCATACGAGATGCTGTAGTGACTGGAGTTCAGACGTGTGCTCTTCCGATCTGATGTCCACGAGGTCTCT | | | IT043 | | | TACAGC | | [24] |
| Barseq_P2_IT 044 | Reverse primer for Barseq, index IT044 | CAAGCAGAAGACGGCATACGAGATATTATAGTGACTGGAGTTCAGACGTGTGCTCTTCCGATCTGATGTCCACGAGGTCTCT | | | IT044 | | | TATAAT | | [24] |
| Barseq_P2_IT 045 | Reverse primer for Barseq, index IT045 | CAAGCAGAAGACGGCATACGAGATGAATGAGTGACTGGAGTTCAGACGTGTGCTCTTCCGATCTGATGTCCACGAGGTCTCT | | | IT045 | | | TCATTC | | [24] |
| Barseq_P2_IT 046 | Reverse primer for Barseq, index IT046 | CAAGCAGAAGACGGCATACGAGATTCGGGAGTGACTGGAGTTCAGACGTGTGCTCTTCCGATCTGATGTCCACGAGGTCTCT | | | IT046 | | | TCCCGA | | [24] |
| Barseq_P2_IT 047 | Reverse primer for Barseq, index IT047 | CAAGCAGAAGACGGCATACGAGATCTTCGAGTGACTGGAGTTCAGACGTGTGCTCTTCCGATCTGATGTCCACGAGGTCTCT | | | IT047 | | | TCGAAG | | [24] |
| Barseq_P2_IT 048 | Reverse primer for Barseq, index IT048 | CAAGCAGAAGACGGCATACGAGATTGCCGAGTGACTGGAGTTCAGACGTGTGCTCTTCCGATCTGATGTCCACGAGGTCTCT | | | IT048 | | | TCGGCA | | [24] |
| Barseq_P2_IT 049 | Reverse primer for Barseq, index IT049 | CAAGCAGAAGACGGCATACGAGATATGTTTGTGACTGGAGTTCAGACGTGTGCTCTTCCGATCTGATGTCCACGAGGTCTCT | | | IT049 | | | AAACAT | | [24] |
| Barseq_P2_IT 050 | Reverse primer for Barseq, index IT050 | CAAGCAGAAGACGGCATACGAGATTGCTTTGTGACTGGAGTTCAGACGTGTGCTCTTCCGATCTGATGTCCACGAGGTCTCT | | | IT050 | | | AAAGCA | | [24] |
| Barseq_P2_IT 051 | Reverse primer for Barseq, index IT051 | CAAGCAGAAGACGGCATACGAGATGCATTTGTGACTGGAGTTCAGACGTGTGCTCTTCCGATCTGATGTCCACGAGGTCTCT | | | IT051 | | | AAATGC | | [24] |
| Barseq_P2_IT 052 | Reverse primer for Barseq, index IT052 | CAAGCAGAAGACGGCATACGAGATTTTGTTGTGACTGGAGTTCAGACGTGTGCTCTTCCGATCTGATGTCCACGAGGTCTCT | | | IT052 | | | AACAAA | | [24] |
| Barseq_P2_IT 053 | Reverse primer for Barseq, index IT053 | CAAGCAGAAGACGGCATACGAGATCAAGTTGTGACTGGAGTTCAGACGTGTGCTCTTCCGATCTGATGTCCACGAGGTCTCT | | | IT053 | | | AACTTG | | [24] |
| Barseq_P2_IT 054 | Reverse primer for Barseq, index IT054 | CAAGCAGAAGACGGCATACGAGATAGTCTTGTGACTGGAGTTCAGACGTGTGCTCTTCCGATCTGATGTCCACGAGGTCTCT | | | IT054 | | | AAGACT | | [24] |
| Barseq_P2_IT 055 | Reverse primer for Barseq, index IT055 | CAAGCAGAAGACGGCATACGAGATTCGCTTGTGACTGGAGTTCAGACGTGTGCTCTTCCGATCTGATGTCCACGAGGTCTCT | | | IT055 | | | AAGCGA | | [24] |
| Barseq_P2_IT 056 | Reverse primer for Barseq, index IT056 | CAAGCAGAAGACGGCATACGAGATGTCCTTGTGACTGGAGTTCAGACGTGTGCTCTTCCGATCTGATGTCCACGAGGTCTCT | | | IT056 | | | AAGGAC | | [24] |
| Barseq_P2_IT 057 | Reverse primer for Barseq, index IT057 | CAAGCAGAAGACGGCATACGAGATCCTATTGTGACTGGAGTTCAGACGTGTGCTCTTCCGATCTGATGTCCACGAGGTCTCT | | | IT057 | | | AATAGG | | [24] |
| Barseq_P2_IT 058 | Reverse primer for Barseq, index IT058 | CAAGCAGAAGACGGCATACGAGATGTTTGTGTGACTGGAGTTCAGACGTGTGCTCTTCCGATCTGATGTCCACGAGGTCTCT | | | IT058 | | | ACAAAC | | [24] |
| Barseq_P2_IT 059 | Reverse primer for Barseq, index IT059 | CAAGCAGAAGACGGCATACGAGATAGATGTGTGACTGGAGTTCAGACGTGTGCTCTTCCGATCTGATGTCCACGAGGTCTCT | | | IT059 | | | ACATCT | | [24] |
| Barseq_P2_IT 060 | Reverse primer for Barseq, index IT060 | CAAGCAGAAGACGGCATACGAGATCTGGGTGTGACTGGAGTTCAGACGTGTGCTCTTCCGATCTGATGTCCACGAGGTCTCT | | | IT060 | | | ACCCAG | | [24] |
| Barseq_P2_IT 061 | Reverse primer for Barseq, index IT061 | CAAGCAGAAGACGGCATACGAGATGCCGGTGTGACTGGAGTTCAGACGTGTGCTCTTCCGATCTGATGTCCACGAGGTCTCT | | | IT061 | | | ACCGGC | | [24] |
| Barseq_P2_IT 062 | Reverse primer for Barseq, index IT062 | CAAGCAGAAGACGGCATACGAGATTATCGTGTGACTGGAGTTCAGACGTGTGCTCTTCCGATCTGATGTCCACGAGGTCTCT | | | IT062 | | | ACGATA | | [24] |
| Barseq_P2_IT 063 | Reverse primer for Barseq, index IT063 | CAAGCAGAAGACGGCATACGAGATGAGAGTGTGACTGGAGTTCAGACGTGTGCTCTTCCGATCTGATGTCCACGAGGTCTCT | | | IT063 | | | ACTCTC | | [24] |
| Barseq_P2_IT 064 | Reverse primer for Barseq, index IT064 | CAAGCAGAAGACGGCATACGAGATTCTTCTGTGACTGGAGTTCAGACGTGTGCTCTTCCGATCTGATGTCCACGAGGTCTCT | | | IT064 | | | AGAAGA | | [24] |
| Barseq_P2_IT 065 | Reverse primer for Barseq, index IT065 | CAAGCAGAAGACGGCATACGAGATCTATCTGTGACTGGAGTTCAGACGTGTGCTCTTCCGATCTGATGTCCACGAGGTCTCT | | | IT065 | | | AGATAG | | [24] |
| Barseq_P2_IT 066 | Reverse primer for Barseq, index IT066 | CAAGCAGAAGACGGCATACGAGATGATGCTGTGACTGGAGTTCAGACGTGTGCTCTTCCGATCTGATGTCCACGAGGTCTCT | | | IT066 | | | AGCATC | | [24] |
| Barseq_P2_IT 067 | Reverse primer for Barseq, index IT067 | CAAGCAGAAGACGGCATACGAGATAGCGCTGTGACTGGAGTTCAGACGTGTGCTCTTCCGATCTGATGTCCACGAGGTCTCT | | | IT067 | | | AGCGCT | | [24] |
| Barseq_P2_IT 068 | Reverse primer for Barseq, index IT068 | CAAGCAGAAGACGGCATACGAGATCGGCCTGTGACTGGAGTTCAGACGTGTGCTCTTCCGATCTGATGTCCACGAGGTCTCT | | | IT068 | | | AGGCCG | | [24] |
| Barseq_P2_IT 069 | Reverse primer for Barseq, index IT069 | CAAGCAGAAGACGGCATACGAGATCCGTATGTGACTGGAGTTCAGACGTGTGCTCTTCCGATCTGATGTCCACGAGGTCTCT | | | IT069 | | | ATACGG | | [24] |
| Barseq_P2_IT 070 | Reverse primer for Barseq, index IT070 | CAAGCAGAAGACGGCATACGAGATTAGGATGTGACTGGAGTTCAGACGTGTGCTCTTCCGATCTGATGTCCACGAGGTCTCT | | | IT070 | | | ATCCTA | | [24] |
| Barseq_P2_IT 071 | Reverse primer for Barseq, index IT071 | CAAGCAGAAGACGGCATACGAGATATAGATGTGACTGGAGTTCAGACGTGTGCTCTTCCGATCTGATGTCCACGAGGTCTCT | | | IT071 | | | ATCTAT | | [24] |
| Barseq_P2_IT 072 | Reverse primer for Barseq, index IT072 | CAAGCAGAAGACGGCATACGAGATGCGTGGGTGACTGGAGTTCAGACGTGTGCTCTTCCGATCTGATGTCCACGAGGTCTCT | | | IT072 | | | CCACGC | | [24] |
| Barseq_P2_IT 073 | Reverse primer for Barseq, index IT073 | CAAGCAGAAGACGGCATACGAGATCATGGGGTGACTGGAGTTCAGACGTGTGCTCTTCCGATCTGATGTCCACGAGGTCTCT | | | IT073 | | | CCCATG | | [24] |
| Barseq_P2_IT 074 | Reverse primer for Barseq, index IT074 | CAAGCAGAAGACGGCATACGAGATTTGCGGGTGACTGGAGTTCAGACGTGTGCTCTTCCGATCTGATGTCCACGAGGTCTCT | | | IT074 | | | CCGCAA | | [24] |
| Barseq_P2_IT 075 | Reverse primer for Barseq, index IT075 | CAAGCAGAAGACGGCATACGAGATCTAAGGGTGACTGGAGTTCAGACGTGTGCTCTTCCGATCTGATGTCCACGAGGTCTCT | | | IT075 | | | CCTTAG | | [24] |
| Barseq_P2_IT 076 | Reverse primer for Barseq, index IT076 | CAAGCAGAAGACGGCATACGAGATTTCTCGGTGACTGGAGTTCAGACGTGTGCTCTTCCGATCTGATGTCCACGAGGTCTCT | | | IT076 | | | CGAGAA | | [24] |
| Barseq_P2_IT 077 | Reverse primer for Barseq, index IT077 | CAAGCAGAAGACGGCATACGAGATCAGCAGGTGACTGGAGTTCAGACGTGTGCTCTTCCGATCTGATGTCCACGAGGTCTCT | | | IT077 | | | CTGCTG | | [24] |
| Barseq_P2_IT 078 | Reverse primer for Barseq, index IT078 | CAAGCAGAAGACGGCATACGAGATGGTTTCGTGACTGGAGTTCAGACGTGTGCTCTTCCGATCTGATGTCCACGAGGTCTCT | | | IT078 | | | GAAACC | | [24] |
| Barseq_P2_IT 079 | Reverse primer for Barseq, index IT079 | CAAGCAGAAGACGGCATACGAGATTTATTCGTGACTGGAGTTCAGACGTGTGCTCTTCCGATCTGATGTCCACGAGGTCTCT | | | IT079 | | | GAATAA | | [24] |
| Barseq_P2_IT 080 | Reverse primer for Barseq, index IT080 | CAAGCAGAAGACGGCATACGAGATTCCGTCGTGACTGGAGTTCAGACGTGTGCTCTTCCGATCTGATGTCCACGAGGTCTCT | | | IT080 | | | GACGGA | | [24] |
| Barseq_P2_IT 081 | Reverse primer for Barseq, index IT081 | CAAGCAGAAGACGGCATACGAGATTATATCGTGACTGGAGTTCAGACGTGTGCTCTTCCGATCTGATGTCCACGAGGTCTCT | | | IT081 | | | GATATA | | [24] |
| Barseq_P2_IT 082 | Reverse primer for Barseq, index IT082 | CAAGCAGAAGACGGCATACGAGATAGCATCGTGACTGGAGTTCAGACGTGTGCTCTTCCGATCTGATGTCCACGAGGTCTCT | | | IT082 | | | GATGCT | | [24] |
| Barseq_P2_IT 083 | Reverse primer for Barseq, index IT083 | CAAGCAGAAGACGGCATACGAGATCCTTGCGTGACTGGAGTTCAGACGTGTGCTCTTCCGATCTGATGTCCACGAGGTCTCT | | | IT083 | | | GCAAGG | | [24] |
| Barseq_P2_IT 084 | Reverse primer for Barseq, index IT084 | CAAGCAGAAGACGGCATACGAGATAAGTGCGTGACTGGAGTTCAGACGTGTGCTCTTCCGATCTGATGTCCACGAGGTCTCT | | | IT084 | | | GCACTT | | [24] |
| Barseq_P2_IT 085 | Reverse primer for Barseq, index IT085 | CAAGCAGAAGACGGCATACGAGATTAAGGCGTGACTGGAGTTCAGACGTGTGCTCTTCCGATCTGATGTCCACGAGGTCTCT | | | IT085 | | | GCCTTA | | [24] |
| Barseq_P2_IT 086 | Reverse primer for Barseq, index IT086 | CAAGCAGAAGACGGCATACGAGATTGGAGCGTGACTGGAGTTCAGACGTGTGCTCTTCCGATCTGATGTCCACGAGGTCTCT | | | IT086 | | | GCTCCA | | [24] |
| Barseq_P2_IT 087 | Reverse primer for Barseq, index IT087 | CAAGCAGAAGACGGCATACGAGATTGTGCCGTGACTGGAGTTCAGACGTGTGCTCTTCCGATCTGATGTCCACGAGGTCTCT | | | IT087 | | | GGCACA | | [24] |
| Barseq_P2_IT 088 | Reverse primer for Barseq, index IT088 | CAAGCAGAAGACGGCATACGAGATCAGGCCGTGACTGGAGTTCAGACGTGTGCTCTTCCGATCTGATGTCCACGAGGTCTCT | | | IT088 | | | GGCCTG | | [24] |
| Barseq_P2_IT 089 | Reverse primer for Barseq, index IT089 | CAAGCAGAAGACGGCATACGAGATGGTAGAGTGACTGGAGTTCAGACGTGTGCTCTTCCGATCTGATGTCCACGAGGTCTCT | | | IT089 | | | TCTACC | | [24] |
| Barseq_P2_IT 090 | Reverse primer for Barseq, index IT090 | CAAGCAGAAGACGGCATACGAGATCATTCAGTGACTGGAGTTCAGACGTGTGCTCTTCCGATCTGATGTCCACGAGGTCTCT | | | IT090 | | | TGAATG | | [24] |
| Barseq_P2_IT 091 | Reverse primer for Barseq, index IT091 | CAAGCAGAAGACGGCATACGAGATATGGCAGTGACTGGAGTTCAGACGTGTGCTCTTCCGATCTGATGTCCACGAGGTCTCT | | | IT091 | | | TGCCAT | | [24] |
| Barseq_P2_IT 092 | Reverse primer for Barseq, index IT092 | CAAGCAGAAGACGGCATACGAGATCCAGCAGTGACTGGAGTTCAGACGTGTGCTCTTCCGATCTGATGTCCACGAGGTCTCT | | | IT092 | | | TGCTGG | | [24] |
| Barseq_P2_IT 093 | Reverse primer for Barseq, index IT093 | CAAGCAGAAGACGGCATACGAGATGCGCCAGTGACTGGAGTTCAGACGTGTGCTCTTCCGATCTGATGTCCACGAGGTCTCT | | | IT093 | | | TGGCGC | | [24] |
| Barseq_P2_IT 094 | Reverse primer for Barseq, index IT094 | CAAGCAGAAGACGGCATACGAGATTTCGAAGTGACTGGAGTTCAGACGTGTGCTCTTCCGATCTGATGTCCACGAGGTCTCT | | | IT094 | | | TTCGAA | | [24] |
| Barseq_P2_IT 095 | Reverse primer for Barseq, index IT095 | CAAGCAGAAGACGGCATACGAGATGGAGAAGTGACTGGAGTTCAGACGTGTGCTCTTCCGATCTGATGTCCACGAGGTCTCT | | | IT095 | | | TTCTCC | | [24] |
| Barseq_P2_IT 096 | Reverse primer for Barseq, index IT096 | CAAGCAGAAGACGGCATACGAGATAAACCTGTGACTGGAGTTCAGACGTGTGCTCTTCCGATCTGATGTCCACGAGGTCTCT | | | IT096 | | | AGGTTT | | [24] |
| **Oligos used for RB-TnSeq** | | | | | | | | | | |
| **Oligo name** | **Notes** | **Sequence** | | | **Index name** | | | **Index** | | **Source** |
| MOD2_TruSeq | Y-adapter for ligation to genomic DNA fragments (1/2) | /5'P/GATCGGAAGAGCACACGTCTGAACTCCAGTCA | | |  | | |  | | [24] |
| Mod2_TS_Univ | Y-adapter for ligation to genomic DNA fragments (2/2) | ACGCTCTTCCGATC*T | | |  | | |  | | [24] |
| Nspacer_barseq_universal | Tn-specific primer for amplification of Tn-seq library | ATGATACGGCGACCACCGAGATCTACACTCTTTCCCTACACGACGCTCTTCCGATCTNNNNNNGATGTCCACGAGGTCT | | |  | | |  | | [24] |
| P7_i6 | Adapter-specific primer for amplification of RB-TnSeq library, indexed | CAAGCAGAAGACGGCATACGAGATATTGGCGTGACTGGAGTTCAGACGTGTGCTCTTCCGATCT | | | i6 | | | GCCAAT | | [24] |
