# Supplementary material for: Construction and characterization of a genome-scale ordered mutant collection of Bacteroides thetaiotaomicron
Source: BMC Biol. 2022 Dec 17;20:285. doi: 10.1186/s12915-022-01481-2 (PMC9758874; doi:10.1186/s12915-022-01481-2)
Supplement: Supplementary file 9 — Additional file 9: Table S3. Program settings for the inoculation of growth curves. [file 12915_2022_1481_MOESM9_ESM.docx]

**Table S3: Program settings for the inoculation of growth curves.**

| **Setting** | **Value** |
| --- | --- |
| Volume | 2 µL |
| Pipetting tool: | TM_50_8 |
| Transfer type | Pipette |
| Use filter tips | Yes |
| Change tips | Before aspirating from a new source |
| Aspiration | Aspirate from defined height: 10.0 mm from bottom. No additional movement. |
| Dispensing | Dispense from defined height: 2.0 mm from bottom. No additional movement. |
| Mix before aspirating | No |
| Mix after dispensing | Yes |
| Standard liquid type | Protein |
| Change parameter | Yes (modified) |
| Aspiration speed | 11 mm/s |
| Dispensing speed | 11 mm/s |
| Blow delay | 0 ms |
| Blow speed | 11 mm/s |
| Immersion depth aspiration | -3 mm |
| Immersion depth dispense | 3 mm |
| Initial stroke | 0 % |
| Blow movement | 0 % |
| Prewetting | 0 cycles |
| Dispense mix: number of cycles | 1 |
| Dispense mix: speed | 11 mm/s |
| Dispense mix: volume | 40 µL |
| Dispense mix: fixed height | No |
